# Resistance training rejuvenates aging skin by reducing circulating inflammatory factors and enhancing dermal extracellular matrices

**DOI:** 10.1038/s41598-023-37207-9

**Published:** 2023-06-23

**Authors:** Shu Nishikori, Jun Yasuda, Kao Murata, Junya Takegaki, Yasuko Harada, Yuki Shirai, Satoshi Fujita

**Affiliations:** 1 Faculty of Sport and Health Science, Ritsumeikan University, 1-1-1 Nojihigashi, Kusatsu, Japan; 2 Frontier Research Center, POLA Chemical Industries, Inc., 560 Kashio-cho, Totsuka-ku, Yokohama, Japan

**Keywords:** Molecular biology, Physiology

## Abstract

Aerobic training (AT) is suggested to be an effective anti-aging strategy for skin aging. However, the respective effects of resistance training (RT) have not been studied. Therefore, we compared the effects of AT and RT on skin aging in a 16-week intervention in 61 healthy sedentary middle-aged Japanese women. Data from 56 women were available for analysis. Both interventions significantly improved skin elasticity and upper dermal structure, and RT also improved dermal thickness. After the training intervention, expression of dermal extracellular matrix-related genes was increased in normal human primary dermal fibroblasts. AT and RT had different effects on circulating levels of factors, such as cytokines, hormones in serum, and metabolites, and RT increased dermal biglycan (*BGN*). To our knowledge, this is the first report to show different effects of AT and RT on skin aging and identify the key factors involved in RT-induced skin rejuvenation.

## Introduction

Exercise is a key strategy for achieving a longer healthy lifespan because it has beneficial effects on physical and physiological health. For example, exercise is known to reverse age-related deterioration in neurogenesis and cognitive function in the brain^1–3^ and improve stem cell function and muscular regeneration in muscle^4,5^. However, the effects of exercise on skin aging are poorly understood, although one study showed that aerobic exercise stimulates release of interleukin 15 (IL-15), which regulates skin aging by enhancing mitochondrial biogenesis in the skin^6^.

The skin is the largest organ in the body and acts as the primary barrier against infection and disease. Moreover, the skin constitutes neuroendocrine system, so the skin is not only the target of neuroendocrine factors but also a source of hormones and neurotransmitters in response to extrinsic and local stress^7,8^. Skin aging is associated with a deterioration in the dermis that results from extracellular matrix (ECM) degradation and is caused by extrinsic factors, such as sun exposure and air pollution^9–11^, and intrinsic factors, such as age-related hormonal changes, neuroendocrine system^12^ and increased levels of inflammatory cytokines; the cytokine changes are referred to as the senescence-associated secretory phenotype^13^. In addition, dermal thickness decreases with aging and ECM degradation^14^.

Exercise training alters the circulating levels of cytokines and hormones^15^, and these changes may be involved in the anti-aging effects of exercise. Recently, research has focused on myokines, i.e., cytokines produced by skeletal muscle cells that are often released into the circulation, as mediators between exercise and various beneficial effects of exercise on health^16,17^ because the secretion of myokines, including IL-15, is mainly induced by exercise. Interestingly, aerobic training (AT) and resistance training (RT) have different effects on circulating levels of various factors^18,19^, and consequently, we hypothesized that they may have different effects on skin aging.

**Figure 1:**
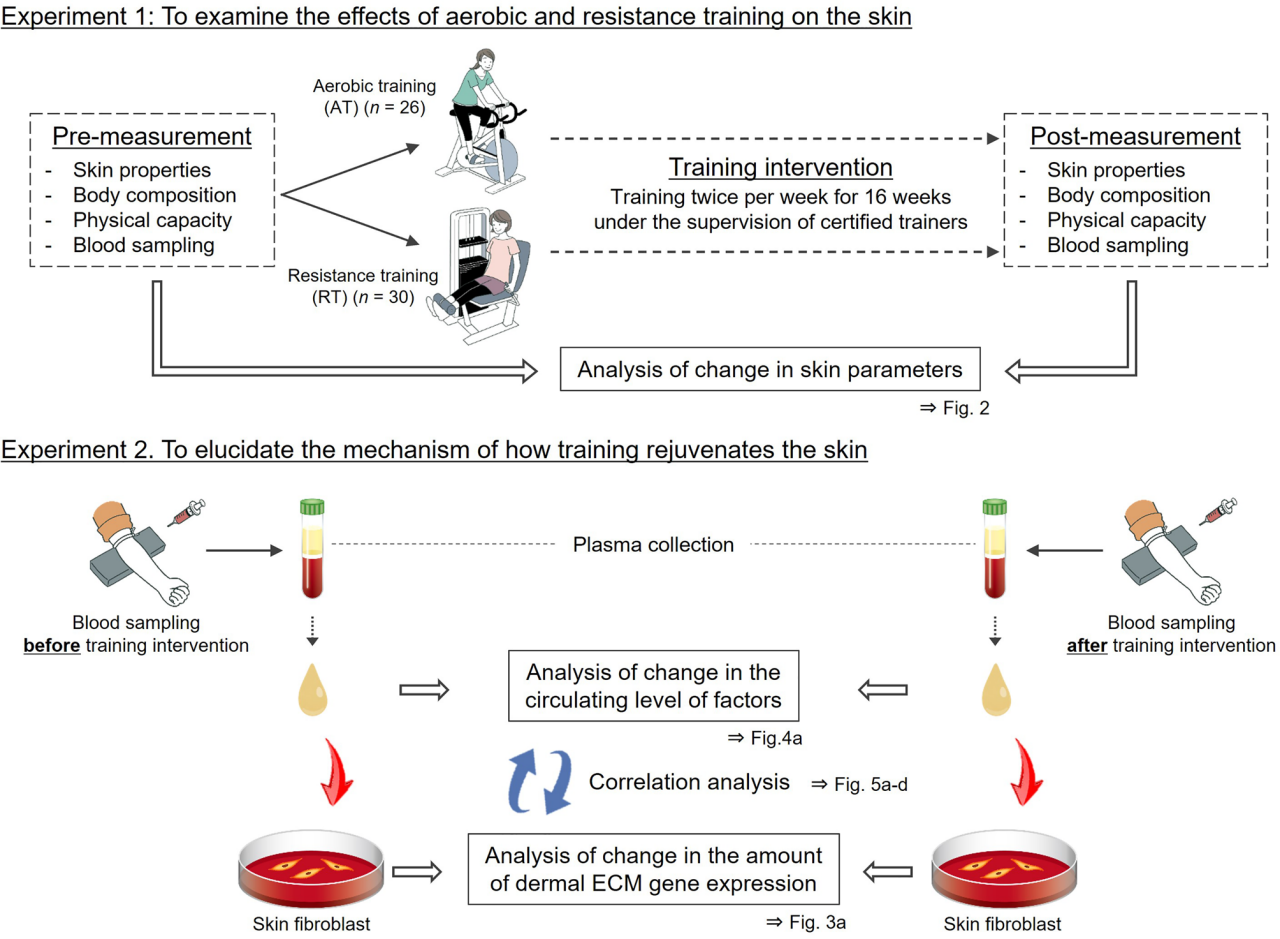
Schematic illustration of the training intervention study. *ECM* extracellular matrix. The illustrations were drawn by an illustrator from LES BANC CO., Ltd. (Tokyo, Japan), and the copyrights were transferred to POLA Chemical Industries, Inc.

Therefore, to compare the effects of AT and RT on skin aging, we performed a 16-week, randomized study in 61 healthy sedentary middle-aged Japanese women and measured circulating levels of various factors in blood samples taken from participants before and after the training intervention. In parallel, plasma samples were added to normal human primary dermal fibroblasts (NHDFs), and the expression of dermal ECM-related genes was quantified (Fig. 1).

## Results

### Study participants

Of the 61 healthy sedentary middle-aged Japanese women recruited into the study, 5 dropped out prematurely. Data from the 56 women who completed the study (AT group, *n* = 26; RT group, *n* = 30) were included in the analyses.

### Effects of AT and RT on body composition, physical capacity, and skin properties

At baseline, no significant differences were found between the AT and RT groups in participant characteristics such as age, dietary intake, skin aging property, body composition, and physical capacity (Extended Data 1).

**Figure 2:**
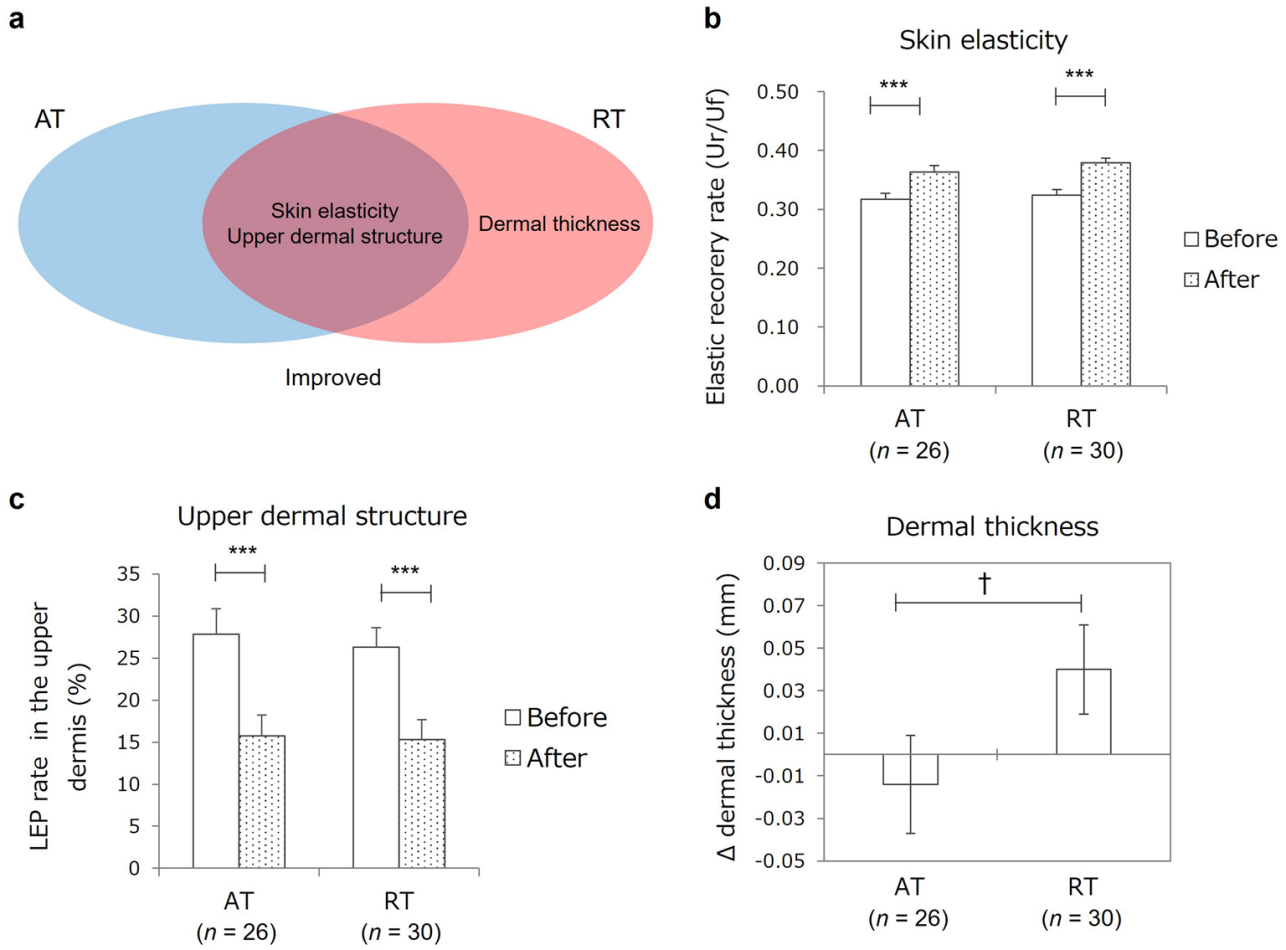
Effects of aerobic training and resistance training on skin aging parameters. (**a**) Venn diagram of skin parameters improved by aerobic training (AT) and resistance training (RT) interventions. Skin parameters in the blue and red elliptical areas were improved by the AT or RT intervention, respectively, and skin parameters in the overlapping area were improved by both interventions. (**b**,**c**) Effects of AT and RT on skin elasticity (**b**) and upper dermal structure (**c**). Elastic recovery rate (Ur/Uf) and rate of low echogenic pixels (LEPs) in the upper dermis were measured as parameters of skin elasticity and upper dermal structure, respectively. (**d**) Differential effect of AT and RT on dermal thickness. Change (Δ) in dermal thickness was calculated as the difference between dermal thickness before and after the training intervention. Columns and bars indicate mean and standard error (SE). Statistical analyses of intragroup difference (**b**,**c**) and differences in Δdermal thickness (**d**) were performed with two-sided paired *t* tests and analysis of co-variance with adjustment for the baseline value of dermal thickness measured before the training intervention, respectively. ***p < 0.001; ^†^p < 0.10. *AT* aerobic training, *LEP* low echogenic pixel, *RT* resistance training.

The AT intervention significantly decreased body weight and body mass index (BMI) and significantly improved peak oxygen uptake (VO_2peak_) as a measure of aerobic capacity, and the RT intervention significantly increased lean soft tissue mass and 1-repetition maximum as a measure of muscular strength (Table 1). Skin elasticity and upper dermal structure, assessed as the rate of low echogenic pixels (LEPs), improved significantly in both groups (Fig. 2a–c, Table 1), and dermal thickness increased in the RT group (Fig. 2d, Extended Data 2).

**Table 1:** Effects of aerobic training and resistance training on skin aging parameters, body composition, and physical capacity.

	All (*n* = 56)			AT (*n* = 26)			RT (*n* = 30)		
	Before	After	*p* value	Before	After	*p* value	Before	After	*p* value
Skin aging parameters									
Skin elasticity, Ur/Uf	0.32 ± 0.01	0.37 ± 0.01	< 0.001	0.32 ± 0.01	0.36 ± 0.01	< 0.001	0.32 ± 0.01	0.38 ± 0.01	< 0.001
Dermal thickness, mm	1.74 ± 0.02	1.75 ± 0.02	0.43	1.71 ± 0.04	1.71 ± 0.02	0.93	1.77 ± 0.03	1.79 ± 0.03	0.20
Upper dermal LEP, %	27.0 ± 1.9	15.5 ± 1.7	< 0.001	27.9 ± 3.0	15.8 ± 2.5	< 0.001	26.3 ± 2.3	15.3 ± 2.3	< 0.001
Body composition									
Body weight, kg	54.4 ± 0.8	54.0 ± 0.8	< 0.05	54.2 ± 1.3	53.5 ± 1.3	< 0.05	54.6 ± 1.1	54.5 ± 1.1	0.76
BMI, kg/m^2^	21.1 ± 0.2	20.9 ± 0.2	< 0.10	21.0 ± 0.3	20.8 ± 0.3	< 0.05	21.1 ± 0.3	21.1 ± 0.3	0.91
AppLTM, kg	15.4 ± 0.2	15.5 ± 0.2	< 0.05	15.4 ± 0.4	15.4 ± 0.4	0.93	15.4 ± 0.3	15.7 ± 0.3	< 0.01
TotalLTM, kg	36.5 ± 0.5	36.7 ± 0.5	< 0.10	36.7 ± 0.8	36.6 ± 0.8	0.72	36.4 ± 0.6	36.8 ± 0.6	< 0.01
Body fat, kg	15.6 ± 0.6	15.3 ± 0.6	< 0.10	15.2 ± 0.8	14.9 ± 0.8	0.19	15.9 ± 0.8	15.6 ± 0.8	0.31
BMC, kg	2.28 ± 0.04	2.27 ± 0.04	0.10	2.25 ± 0.06	2.24 ± 0.06	0.22	2.31 ± 0.05	2.30 ± 0.05	0.28
Physical capacity									
VO_2peak,_ mL/kg/min	28.0 ± 0.6	28.9 ± 0.6	< 0.10	27.1 ± 0.8	28.5 ± 0.7	< 0.05	28.7 ± 1.0	29.3 ± 0.8	0.50
1-RM, kg									
Chest press	11.5 ± 0.4	14.9 ± 0.6	< 0.001	11.2 ± 0.6	11.6 ± 0.6	0.10	11.8 ± 0.6	17.7 ± 0.7	< 0.001
Shoulder press	15.7 ± 0.5	20.0 ± 0.8	< 0.001	14.9 ± 0.7	15.6 ± 0.8	0.07	16.3 ± 0.7	23.8 ± 0.9	< 0.001
Arm curl	15.2 ± 0.5	18.9 ± 0.7	< 0.001	15.0 ± 0.8	15.6 ± 0.7	0.24	15.4 ± 0.5	21.7 ± 0.8	< 0.001
Rowing	22.1 ± 0.5	24.6 ± 0.7	< 0.001	21.7 ± 1.0	20.9 ± 0.8	0.11	22.5 ± 0.6	27.8 ± 0.8	< 0.001
Leg curl	43.4 ± 1.2	50.3 ± 1.7	< 0.001	42.0 ± 2.0	42.8 ± 2.0	0.56	44.6 ± 1.4	56.7 ± 2.0	< 0.001
Leg extension	68.2 ± 2.4	77.2 ± 2.5	< 0.001	65.5 ± 3.7	68.8 ± 3.5	0.11	70.5 ± 3.0	84.7 ± 3.0	< 0.001

Values show the means ± standard errors (SEs). Statistical analyses were performed with two-sided paired *t* tests to compare parameters measured before and after the training intervention.

*AppLTM* appendicular lean soft tissue mass, *AT* aerobic training, *BMC* bone mineral content, *BMI* body mass index, *LEP* low echogenic pixel, *RT* resistance training, *TotalLTM* total lean soft tissue mass, *VO*_*2peak*_ peak oxygen uptake, *1-RM* one-repetition maximum.

### Effects of blood plasma after AT and RT on expression of dermal ECM-related genes

**Figure 3:**
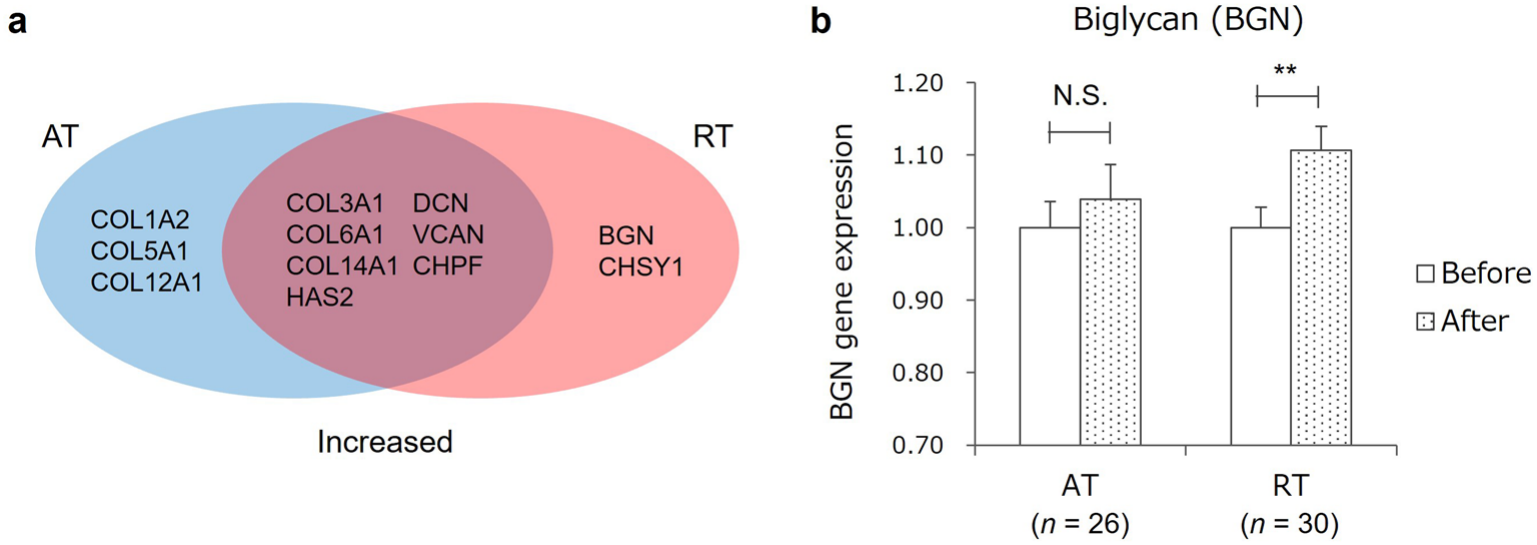
Effects of aerobic training and resistance training on expression of dermal extracellular matrix-related genes in cultured skin fibroblasts. (**a**) Venn diagram of dermal extracellular matrix genes with increased expression after aerobic training (AT) and resistance training (RT). Expression levels of genes in the blue and red elliptical areas were higher in plasma after the AT and RT intervention, respectively. Expression levels of genes in the overlapping area were higher in plasma after both AT and RT. (**b**) Differential effect of AT and RT on *BGN* expression. Columns and bars indicate mean and standard error (SE). Statistical analyses of intragroup differences were performed with two-sided paired *t* tests. **, *p* < 0.01; *N.S.* not significant, *AT* aerobic training, *BGN* biglycan, *CHPF* chondroitin polymerizing factor, *CHSY1* chondroitin sulfate synthase 1, *COL1A2* collagen type I α 2 chain, *COL3A1*, collagen type III α 1 chain, *COL5A1* collagen type V α 1 chain, *COL6A1* collagen type VI α 1 chain, *COL12A1* collagen type XII α 1 chain, *COL14A1* collagen type XIV α 1 chain, *DCN* decorin, *HAS2* hyaluronan synthase 2, *RT* resistance training, *VCAN* versican.

To explore the mechanism by which the training interventions improved dermal aging, plasma from blood sampled at rest before and after the 16-week training intervention was added to cultured NHDFs, and the expression of dermal ECM genes was quantified. In both groups, expression of dermal ECM-related genes such as those encoding collagens (*COL3A1*, *COL6A1*, and *COL14A1*), hyaluronan synthase 2 (*HAS2*), and proteoglycans (decorin [*DCN*], versican [*VCAN*], and chondroitin polymerizing factor [*CHPF*]) was increased in plasma after training compared with before training (Fig. 3a, Table 2). After AT, the expression of other collagen genes, such as *COL1A2*, *COL5A1*, and *COL12A1*, was increased, and after RT, the expression of other proteoglycan-related genes, such as biglycan (*BGN*) and chondroitin sulfate synthase 1 (*CHSY1*) was increased (Fig. 3a,b, Table 2).

**Table 2:** Effects of aerobic training and resistance training on expression level of dermal extracellular matrix-related genes in dermal fibroblasts (measured in plasma from blood sampled from participants before and after the 16-week training intervention).

Gene	All (*n* = 56)				AT (*n* = 26)				RT (*n* = 30)			
	Before	After		*p* value	Before	After		*p* value	Before	After		*p* value
*COL1A1*	0.821 ± 0.025	0.858 ± 0.032	–	0.19	0.857 ± 0.043	0.898 ± 0.053	–	0.37	0.789 ± 0.029	0.823 ± 0.037	–	0.36
*COL1A2*	0.826 ± 0.023	0.865 ± 0.026	↑	< 0.10	0.862 ± 0.034	0.928 ± 0.043	↑	< 0.10	0.795 ± 0.030	0.811 ± 0.027	–	0.60
*COL3A1*	0.895 ± 0.031	0.969 ± 0.033	↑	< 0.01	0.945 ± 0.047	1.040 ± 0.055	↑	< 0.05	0.851 ± 0.040	0.908 ± 0.037	↑	< 0.10
*COL5A1*	0.727 ± 0.018	0.764 ± 0.021	↑	< 0.05	0.757 ± 0.028	0.807 ± 0.035	↑	< 0.10	0.702 ± 0.024	0.727 ± 0.025	–	0.26
*COL6A1*	0.809 ± 0.018	0.908 ± 0.023	↑	< 0.001	0.845 ± 0.025	0.947 ± 0.035	↑	< 0.05	0.778 ± 0.024	0.873 ± 0.028	↑	< 0.001
*COL12A1*	0.981 ± 0.028	1.062 ± 0.032	↑	< 0.01	1.044 ± 0.047	1.159 ± 0.052	↑	< 0.01	0.926 ± 0.030	0.978 ± 0.035	–	0.10
*COL14A1*	0.835 ± 0.023	0.931 ± 0.027	↑	< 0.001	0.887 ± 0.040	0.984 ± 0.042	↑	< 0.05	0.791 ± 0.024	0.885 ± 0.034	↑	< 0.01
*HAS1*	0.092 ± 0.013	0.102 ± 0.017	–	0.52	0.090 ± 0.013	0.115 ± 0.032	–	0.34	0.095 ± 0.021	0.091 ± 0.016	–	0.98
*HAS2*	0.177 ± 0.020	0.239 ± 0.022	↑	< 0.001	0.163 ± 0.018	0.247 ± 0.026	↑	< 0.001	0.190 ± 0.034	0.233 ± 0.035	↑	< 0.01
*DCN*	0.911 ± 0.029	0.994 ± 0.032	↑	< 0.001	0.973 ± 0.043	1.080 ± 0.049	↑	< 0.01	0.857 ± 0.036	0.919 ± 0.036	↑	< 0.10
*BGN*	0.685 ± 0.016	0.736 ± 0.021	↑	< 0.01	0.722 ± 0.026	0.750 ± 0.036	–	0.31	0.653 ± 0.019	0.723 ± 0.024	↑	< 0.001
*VCAN*	0.595 ± 0.017	0.644 ± 0.019	↑	< 0.01	0.635 ± 0.028	0.691 ± 0.034	↑	< 0.05	0.561 ± 0.019	0.604 ± 0.019	↑	< 0.01
*CHPF*	1.062 ± 0.030	1.162 ± 0.037	↑	< 0.001	1.146 ± 0.048	1.252 ± 0.052	↑	< 0.05	0.989 ± 0.035	1.084 ± 0.048	↑	< 0.01
*CHSY1*	1.040 ± 0.042	1.110 ± 0.047	↑	< 0.05	1.106 ± 0.068	1.186 ± 0.071	–	0.17	0.981 ± 0.049	1.044 ± 0.060	↑	< 0.05
*CHSY3*	1.630 ± 0.075	1.653 ± 0.075	–	0.65	1.766 ± 0.121	1.743 ± 0.098	–	0.79	1.513 ± 0.091	1.576 ± 0.111	–	0.31
*ELN*	0.716 ± 0.036	0.638 ± 0.037	↓	< 0.05	0.755 ± 0.052	0.651 ± 0.058	↓	< 0.05	0.682 ± 0.051	0.626 ± 0.047	–	0.24

Values show the means of the relative level of gene expression calculated by the ΔΔCt method (where Ct is the threshold cycle) normalized with glyceraldehyde 3-phosphate dehydrogenase as the housekeeping gene and the control sample ± standard errors (SEs). Statistical analyses for the intragroup difference were performed with two-sided paired *t* tests.

*AT* aerobic training, *BGN* biglycan, *CHPF* chondroitin polymerizing factor, *CHSY1* chondroitin sulfate synthase 1, *CHSY3* chondroitin sulfate synthase 3, *COL1A1* collagen type I α 1 chain, *COL1A2* collagen type I α 2 chain, *COL3A1* collagen type III α 1 chain, *COL5A1* collagen type V α 1 chain, *COL6A1* collagen type VI α 1 chain, *COL12A1* collagen type XII α 1 chain, *COL14A1* collagen type XIV α 1 chain, *DCN* decorin, *ELN* elastin, *HAS1* hyaluronan synthase 1, *HAS2* hyaluronan synthase 2, *RT* resistance training, *VCAN* versican.

### Effects of AT and RT on circulating levels of various factors

**Figure 4:**
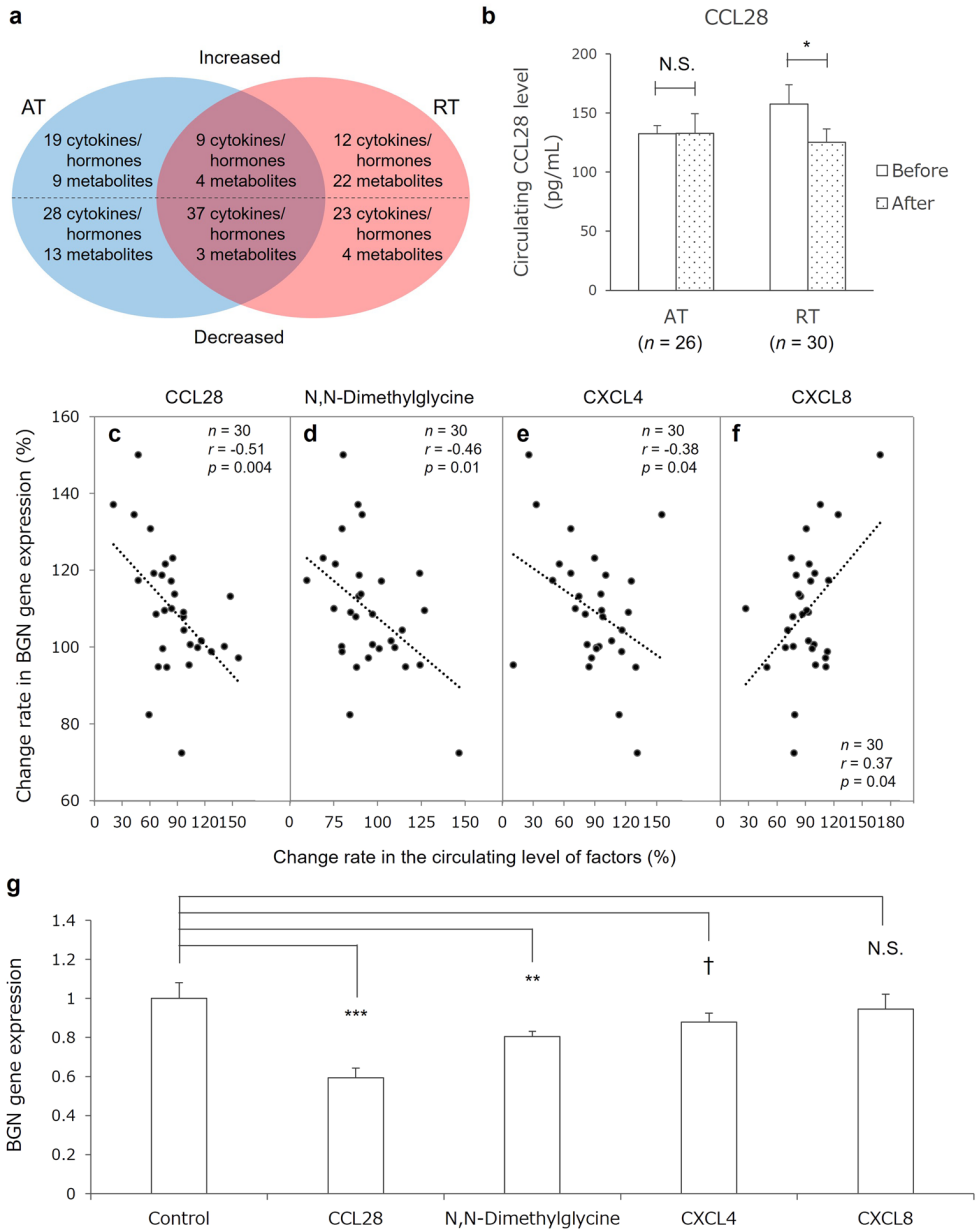
Identification of blood factors affecting biglycan (*BGN*) expression. (**a**) Venn diagram of circulating factors increased or decreased by aerobic training (AT) and resistance training (RT). The circulating level of factors in the blue and red elliptical areas was increased (factors written above the dashed line) or decreased (factors written below the dashed line) by the AT and RT interventions, respectively. The circulating level of factors in the overlapping area was increased (above the line) and decreased (below the line) by both the AT and RT interventions. (**b**) Differential effect of AT and RT on the circulating level of C–C motif chemokine ligand 28 (CCL28). Columns and bars indicate mean and standard error (SE). Statistical analyses for the intragroup difference were performed with two-sided paired *t* tests. *p < 0.05; *N.S.* not significant. (**c**–**f**) Correlation between change rate in biglycan (*BGN*) expression and that of the circulating level of CCL28 (**c**), *N*,*N*-dimethylglycine (**d**), C–X–C motif chemokine 4 (**e**), and C–X–C motif chemokine 8 (**f**). Change in *BGN* expression was calculated as the change of expression in skin fibroblasts cultured with plasma from blood sampled before and after the training intervention. Changes in the circulating levels of factors were calculated as the difference in the circulating level before and after the training intervention. The dots and lines indicate the participants in the RT group and the regression lines, respectively. (**g**) Effects of candidate factors on *BGN* gene expression. The columns and bars indicate mean and standard error (SE). The concentrations were determined from the mean blood concentration in participants before the training intervention. The mean concentrations were as follows: CCL28, 150 pg/mL; *N*,*N*-dimethylglycine, 7 μM; CXCL4, 10 μg/mL; and CXCL8, 10 pg/mL. Statistical analyses were performed with Dunnett’s test. *r* Pearson correlation coefficient, *p < 0.05; **p < 0.01; ***p < 0.001; ^†^p < 0.10; *N.S.* not significant, *AT* aerobic training, *BGN* biglycan, *CCL28* C–C motif chemokine ligand 28, *CXCL4* C–X–C motif chemokine 4, *CXCL8* C–X–C motif chemokine 8, *RT* resistance training.

The circulating level of more than 1480 factors, such as cytokines, serum hormones, and plasma metabolites, was measured in blood sampled before and after the training interventions. A number of circulating factors showed significant changes after the training interventions (Fig. 4a; detailed data are shown in Extended Data 3–8). In the AT group, the circulating level of 28 factors increased, including myokines such as IL-15 and myonectin, and that of 41 factors decreased, including inflammatory factors such as interferon gamma and tumor necrosis-related apoptosis-inducing ligand. In the RT group, the circulating level of 34 factors increased, including myokines such as cathepsin B, C-X-C motif chemokine ligand 8 (CXCL8), and regulated on activation, normal T-cell expressed and secreted increased, and that of 27 factors decreased, including inflammatory factors such as monocyte chemotactic protein 3 and C–C motif chemokine ligand 28 (CCL28) (Fig. 4b).

### Screening of candidate factors affecting expression of biglycan (*BGN*)

To elucidate the mechanism of the RT-specific increase in dermal thickness, we focused on the RT-induced increase in the expression of biglycan (*BGN*) because *BGN*-knockout mice show a thinned dermis phenotype^20^. To screen candidate factors that may affect *BGN* expression, we performed correlation analyses between the change in *BGN* expression and that in circulating levels of other factors. Three factors that showed decreased circulating levels after the RT intervention (CCL28, N,N-dimethylglycine, and C-X-C motif chemokine 4 [CXCL4]) were significantly negatively correlated with the change in *BGN* expression (Fig. 4c–e), suggesting that these factors suppress *BGN* expression. In addition, the increase in the circulating level of CXCL8 after RT significantly positively correlated with the change in *BGN* expression (Fig. 4f), suggesting that CXCL8 enhances *BGN* expression.

### Identification of factors affecting *BGN* expression

To identify factors affecting *BGN* expression, the four candidate factors identified in the above-mentioned screening were individually added to cultured NHDFs, and the expression of *BGN* was quantified. Then, CCL28, N,N-dimethylglycine, and CXCL4 suppressed *BGN* expression (Fig. 4g). Furthermore, these three factors also suppressed the expression level of other dermal ECM-related genes (Table 3).

**Table 3:** Effects of factors identified as affecting biglycan (*BGN*) on expression of other dermal extracellular matrix-related genes.

Gene	CCL28			*N*,*N*-Dimethylglycine			CXCL4		
	*vs* control (1.00)		*p* value	*vs* control (1.00)		*p* value	*vs* control (1.00)		*p* value
*COL1A1*	0.72 ± 0.05	↓	< 0.01	0.89 ± 0.05	↓	< 0.10	0.93 ± 0.14	–	0.47
*COL1A2*	0.79 ± 0.03	↓	< 0.01	0.83 ± 0.08	↓	< 0.05	0.98 ± 0.03	–	0.58
*COL3A1*	0.81 ± 0.06	↓	< 0.05	0.91 ± 0.09	–	0.20	0.82 ± 0.07	↓	< 0.05
*COL5A1*	0.68 ± 0.05	↓	< 0.01	0.74 ± 0.04	↓	< 0.01	1.01 ± 0.05	–	0.88
*COL6A1*	0.94 ± 0.07	–	0.21	0.92 ± 0.03	–	< 0.05	1.00 ± 0.04	–	0.85
*COL12A1*	0.73 ± 0.05	↓	< 0.01	0.86 ± 0.05	↓	< 0.05	1.01 ± 0.12	–	0.91
*COL14A1*	0.73 ± 0.04	↓	< 0.01	0.92 ± 0.20	–	0.57	0.71 ± 0.02	↓	< 0.01
*HAS1*	0.38 ± 0.14	↓	< 0.01	0.23 ± 0.11	↓	< 0.01	1.53 ± 0.18	↑	< 0.05
*HAS2*	0.74 ± 0.05	↓	< 0.01	1.05 ± 0.04	–	0.28	1.32 ± 0.11	↑	< 0.05
*DCN*	0.70 ± 0.07	↓	< 0.01	0.91 ± 0.07	↓	< 0.10	0.85 ± 0.05	↓	< 0.05
*BGN*	0.56 ± 0.05	↓	< 0.01	0.76 ± 0.03	↓	< 0.05	0.83 ± 0.04	↓	< 0.10
*VCAN*	0.83 ± 0.07	↓	< 0.05	0.89 ± 0.09	–	0.12	1.03 ± 0.03	–	0.32
*CHPF*	0.76 ± 0.05	↓	< 0.01	0.84 ± 0.03	↓	< 0.01	1.07 ± 0.02	↑	< 0.05
*CHSY1*	0.72 ± 0.05	↓	< 0.001	0.91 ± 0.03	↓	< 0.05	1.07 ± 0.05	–	0.10
*CHSY3*	0.76 ± 0.04	↓	< 0.01	0.93 ± 0.07	–	0.24	0.96 ± 0.08	–	0.50
*ELN*	0.71 ± 0.02	↓	< 0.001	0.86 ± 0.09	↓	< 0.10	1.09 ± 0.06	–	0.11

Values show the means of the relative gene expression amounts calculated by the ΔΔCt method (where Ct is the threshold cycle) normalized with glyceraldehyde 3-phosphate dehydrogenase as the housekeeping gene and the control sample ± standard errors (SEs). Statistical analyses were performed with two-sided independent *t* tests.

*BGN* byglycan, *CCL28* C–C motif chemokine ligand 28, *CHPF* chondroitin polymerizing factor, *CHSY1* chondroitin sulfate synthase 1, *CHSY3* chondroitin sulfate synthase 3, *COL1A1* collagen type I α 1 chain, *COL1A2* collagen type I α 2 chain, *COL3A1* collagen type III α 1 chain, *COL5A1* collagen type V α 1 chain, *COL6A1* collagen type VI α 1 chain, *COL12A1* collagen type XII α 1 chain, *COL14A1* collagen type XIV α 1 chain, *CXCL4* C–X–C motif chemokine 4, *DCN* decorin, *ELN* elastin, *HAS1* hyaluronan synthase 1, *HAS2* hyaluronan synthase 2, *VCAN* versican.

## Discussion

In this study, we showed that RT counteracts skin aging such as deteriorations in skin elasticity, upper dermal structure and dermal thickness. Our findings suggest that the increase in dermal thickness is a specific effect of RT on the skin and is induced by a decrease in circulating levels of CCL28, N,N-dimethylglycine, and CXCL4 and an associated increase in dermal *BGN* expression. AT also had positive effects on skin elasticity and upper dermal structure, but it did not improve dermal thickness.

AT and RT have different physiological effects on the human body^21,22^ and a meta-analysis indicated that RT promotes muscle hypertrophy more than AT does^21^. The present study also showed a significant increase in lean soft tissue mass (LTM) and 1-repetition maximum in the RT group (Table 1), confirming that this RT intervention was effective. The increase in total LTM in the RT group was 0.44 ± 0.13 kg (95% confidence interval 0.17–1.70), corresponding to a rate of change of 1.2 ± 0.35%. The degree of muscle hypertrophy was reasonable for middle-aged participants, because middle-aged individuals (45–55 years of age, *n* = 20, 50% women) who undertook a 20-week RT program (70% RM, whole body targeted [8 exercises], 3 times per week) experienced 1.1% increase in total LTM^23^. In the AT group, we found a significant decrease in body weight and BMI and a significant increase in VO_2peak_ (Table 1).

A comparison of AT and RT based on the hypothesis that the two types of training have different effects on the skin showed that RT specifically increased dermal thickness, which is known to decrease with aging^14^. In addition, both AT and RT showed same rejuvenating effects on the skin, such as improving elasticity and upper dermal structure, by inducing an increase in dermal ECMs. Elasticity is a general physical property of skin that is known to decrease with aging^24,25^, and the rate of LEP in the upper dermal layer is reported to increase with aging, generating the low echogenic area at the upper dermis called the “aging band” or “subepidermal low echogenic band”^26–28^. Both skin elasticity and upper dermal structure deteriorate because of not only aging but also sun exposure^27,29^, and changes in both are thought to be related to a decrease in dermal ECMs. In fact, the ECM is suggested to be related to the elastic property^30,31^ and ultrasound echogenicity of the dermis^32^.

In aged mice, exercise increased neurogenesis and cognitive function, and glycosylphosphatidylinositol (GPI)-specific phospholipase D1 was identified as an exercise-induced, liver-derived circulating factor that was sufficient to improve hippocampal function^2^. Regarding the skin, aerobic exercise was reported to stimulate mitochondrial biogenesis in skin fibroblasts and increase dermal collagen content, and IL-15 was identified as the muscle-derived mediator of these effects^6^. Our study also found that AT increased the circulating level of IL-15 and improved dermal structure. In this study, we found that the expression of major collagen fibril genes, such as *COL1A2* and *COL3A1*, proteoglycan genes, such as *DCN* and *VCAN*, and *HAS2* was increased after both interventions. These results suggest that training-induced changes in circulating factors increase numerous components of the dermal ECM, leading to improvements in skin elasticity and upper dermal structure.

After the RT intervention, dermal thickness and expression levels of *BGN* and *CHSY1* increased. The relationship between *CHSY1* and dermal thickness is unclear, but *BGN*-knockout mice were reported to show a thinned dermis phenotype^20^, and biglycan levels were found to decrease with aging and sun-exposure^33^; taken together, these findings suggest that increased *BGN* expression could lead to increased dermal thickness. Moreover, the present study identified circulating CCL28, N,N-dimethylglycine, and CXCL4, which were decreased specifically by the RT intervention, as factors that suppressed *BGN* expression. Circulating levels of CCL28 and CXCL4 were reported to be markers of severity of atopic dermatitis and systemic sclerosis, respectively^34,35^. CCR3 and CCR10 are known as receptors for CCL28 and are reported to be expressed on dermal fibroblasts, and the interaction of CCL28 with CCR3 and CCR10 was found to regulate cutaneous wound healing^34,36^. In the present study, CCL28 suppressed the effects of numerous dermal ECM-related genes (Table 3), suggesting that it plays a key role in regulating skin homeostasis and aging. CXCR3, a receptor for CXCL4, is also expressed on fibroblasts, and CXCR3 signaling is related to the cutaneous inflammatory reaction^37,38^.

The circulating level of inflammatory factors is speculated to be enhanced by factors including aging, stress, and physical inactivity^39–41^. In contrast, aerobic exercise is an effective strategy to reduce circulating inflammatory factors^15,42,43^. Taken together, the results of the present study suggest that not only AT but also RT attenuates the circulating level of inflammatory factors and improves dermal aging. In particular, the results indicate that RT increases the thinning dermis and simultaneously reduces circulating levels of CCL28 and CXCL4, which were identified as key inflammatory factors that suppress the expression of *BGN*, thereby helping to improve the thickness of the dermis. But here is a limitation that our conclusion is considered by the following 3 separate results; (1) RT increased dermal thickness, (2) blood plasma after RT increased *BGN* relating to dermal thickness, and (3) circulating inflammatory factors reduced by RT suppressed *BGN* gene expression. Therefore, more direct animal studies modulating circulating inflammatory factors are needed to clearly confirm the skin rejuvenating mechanism of RT.

**Figure 5:**
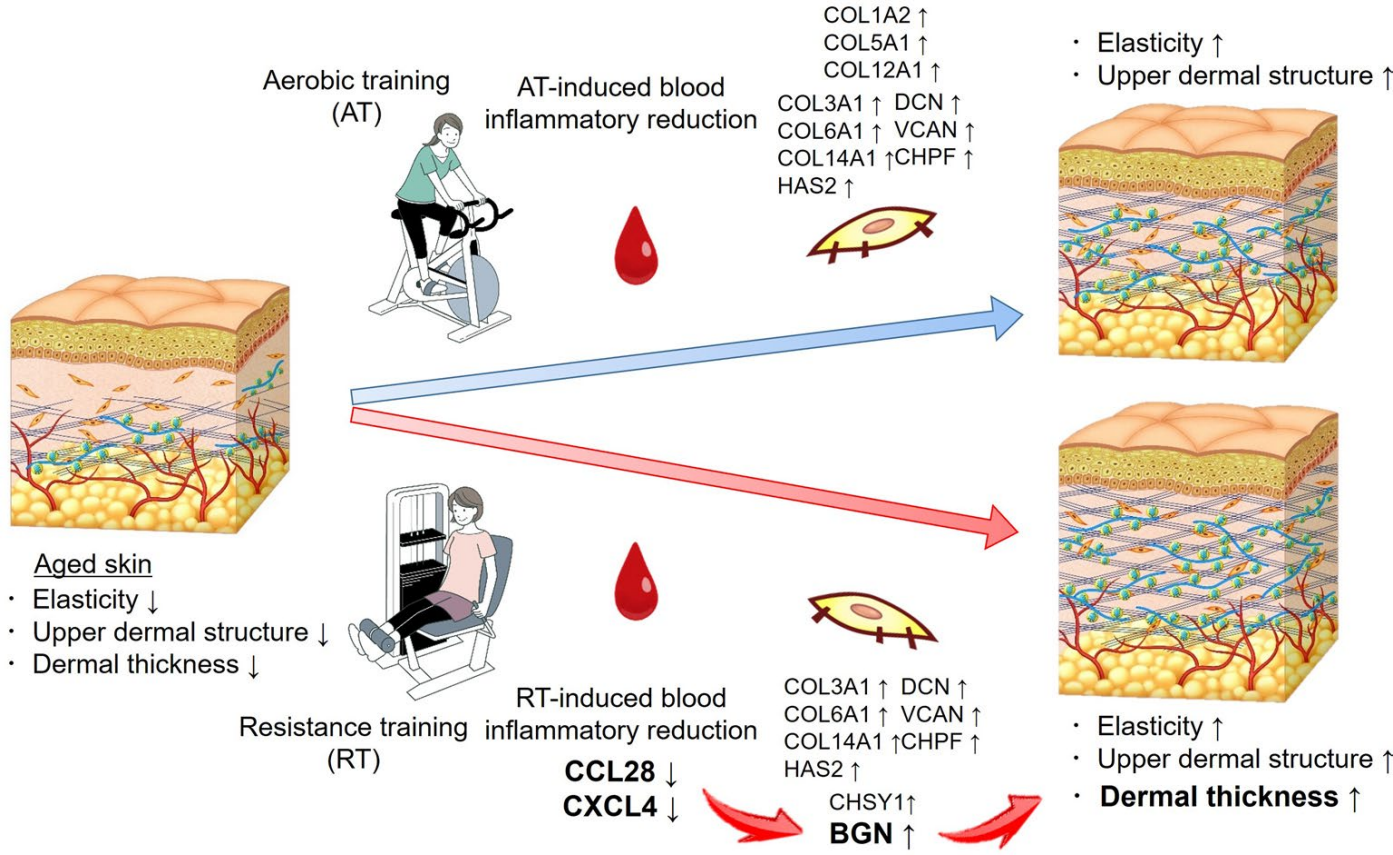
Skin rejuvenating effects of aerobic training and resistance training. *BGN* biglycan, *CCL28* C–C motif chemokine ligand 28, *CHPF* chondroitin polymerizing factor, *CHSY1* chondroitin sulfate synthase 1, *COL1A2* collagen type I α 2 chain, *COL3A1* collagen type III α 1 chain, *COL5A1* collagen type V α 1 chain, *COL6A1* collagen type VI α 1 chain, *COL12A1* collagen type XII α 1 chain, *COL14A1* collagen type XIV α 1 chain, *CXCL4* C–X–C motif chemokine 4, *DCN* decorin, *HAS2* hyaluronan synthase 2, *VCAN* versican. The illustrations were drawn by an illustrator from LES BANC CO., Ltd. (Tokyo, Japan), and the copyrights were transferred to POLA Chemical Industries, Inc.

In conclusion, a 16-week intervention with AT and RT showed that both training interventions counteract skin aging by improving skin elasticity and upper dermal structure. In addition, RT increases dermal thickness by inducing a reduction in circulating levels of CCL28, N,N-dimethylglycine, and CXCL4 and thus suppressing expression of dermal *BGN* (Fig. 5). The study clarified only the mechanism by which RT counteracts age-associated dermal thinning, and the other mechanisms of AT- and RT-driven skin rejuvenation remain to be elucidated.

## Methods

### Study design

A total of 61 healthy sedentary middle-aged Japanese women aged 41–59 years living in Kusatsu, Japan, were recruited into the training intervention study and randomly assigned to the AT group (n = 27) and RT group (n = 34). Randomization sequences were generated in Microsoft Excel with a combination of RAND and RANK functions. For 16 weeks from February to May 2019, participants performed AT or RT twice per week under the supervision of certified trainers in a training room (32 sessions in total). A schematic illustration of the study is shown in Fig. 1, and details of the exercise regimens are provided below.

Strength was assessed regularly to ensure that participants achieved the target workload, i.e., 65–70% of the maximum heart rate for AT and 75–80% of the repetition maximum for RT. Before and after the 16-week training intervention, skin properties, body composition, and physical capacity were evaluated and blood samples were obtained. During the intervention study, participants were asked not to perform any additional exercise, take any supplement and hormone therapy, and change their skincare routines.

The study complied with all relevant ethical regulations. The training intervention study was approved by the ethics committees of Ritsumeikan University, Kusatsu, Japan (BKC-IRB-2018-031-1) and POLA Chemical Industries, Inc., Yokohama, Japan (2018-G-133). The human clinical study was conducted in accordance with the Declaration of Helsinki. All participants were informed about the experimental procedures, possible risks, and benefits of the study, and all of them provided written informed consent.

#### Aerobic capacity and strength assessment

Aerobic capacity and strength were assessed before and after the training intervention. Peak aerobic capacity was estimated as a VO_2peak_ by monitoring oxygen uptake during an incremental cycling test on an electronic bicycle ergometer (Ergomedic 828 E, Monark Exercise, Vansbro, Sweden). The heart rate at which maximal oxygen uptake was reached was set as the peak heart rate. Maximum muscular strength was estimated by 1-repetition maximum strength tests on the following weight-stack machines: leg curl, leg extension, arm curl, rowing, shoulder press, and chest press (Life Fitness, Rosemont, IL, USA).

#### Training program

Participants attended exercise training sessions in the morning or afternoon and were permitted to switch between the two times, and researchers kept a record of all sessions. The AT program consisted of a 3-min warm-up session on an electronic bicycle ergometer (Ergomedic 828 E, Monark Exercise) at 0 watts and 60 rpm, 30 min of the main AT program comprising cycling at 65–70% of the peak heart rate adjusted with pedal load, and a 3-min cooldown at 0 watts and 60 rpm. The RT program consisted of a 5-repetition warm-up session on each machine at 50% of the 1-repetition maximum load, followed by 3 sets of 10 repetitions as the main RT program on the following 6 weight-stack machines: leg curl, leg extension, arm curl, rowing, shoulder press, and chest press (Life Fitness)^44^. The RT loads increased throughout the study, as follows: 50% (sessions 1 and 2), 60% (sessions 3–6), 70% (sessions 7–10), and 75–80% (sessions 11–32); during sessions 11–32, at each session the load was increased by 5% of the load at which the participants previously achieved 10 repetitions in the third set. A 2- to 3-min rest was allowed between sets. A certified trainer supervised all the sessions and, at every session, confirmed whether the weight lifted at each machine was the target load (75–80% of the repetition maximum) and tempo (90 beats per min: 1, concentric; 3, eccentric)^44^.

Participants were asked to refrain from performing any other exercise until completion of the final measurements.

### Measurement of body composition

Body composition was measured by dual-energy X-ray absorptiometry by the same radiological technician in the morning before and after the 16-week training intervention. The dual-energy X-ray absorptiometry apparatus (Lunar Prodigy, GE Healthcare, Chicago, IL, USA) was calibrated at the beginning of each measurement day, and, with participants lying in a supine position, the lean soft tissue mass of the arms, legs, and trunk and fat mass were analyzed by software (enCORE, GE Healthcare) and the total lean soft tissue mass and appendicular lean soft tissue mass were calculated.

### Dietary assessment

The participants were asked to record their dietary intake on the usual 3 days which were either nonconsecutive or consecutive days^45^, before and after the 16-week training intervention. The dietary records included the following instructions:

“Please note your dietary records on 2 weekdays and 1 day of the weekend.”

“Please note all foods you had, including confectionery and beverages.”

“Please take pictures of foods or nutrition facts and note whether the food is cooked or processed before consumption.”

“Please complete your dietary records by referring to the examples provided.”

To improve the accuracy of the dietary assessment, participants were asked to photograph the 3-day record using their phone cameras, which were then collected and confirmed by a registered dietitian via face-to-face interviews with the participants. Before the beginning of the study, all participants attended an explanatory meeting on the methodology of noting dietary records and were asked to maintain their own dietary habits. The data were analyzed using Excel Eiyokun, version 8 (Kenpakusha Co., Tokyo, Japan) based on the Standard Table of Food Composition in Japan-2015.

### Measurement of skin properties

Skin properties were evaluated by the same trained researcher in the morning before the training intervention and within a week of the last training session. After removing makeup and washing their face, participants were acclimatized for 10 min to an ambient condition in a room at a temperature of 20–22 °C and a relative humidity of 50–55%. With the participants lying in a supine position, skin elasticity, dermal echogenicity, dermal thickness, and skin tone at the center of the left cheek were measured. The elastic recovery rate was measured as skin elasticity with a suction device (Cutometer, Courage + Khazaka electronic GmbH, Cologne, Germany) with a 2-mm diameter probe at a reduced pressure of 400 mbar; the measurement was performed by using 2 s of suction followed by 2 s of release^24,25^. The cross-sectional image of the skin was obtained with a 50-MHz ultrasound scanner (DermaScan, Cortex Technology, Hadsund, Denmark), and the number of pixels and LEPs in the upper, middle, and lower dermal layers and the dermal thickness were analyzed in B-mode^26,27^. The amount of LEPs in each dermal layer was used as a parameter for dermal structure. Skin tone was measured with a spectrophotometer (CM-600d, KONICA MINOLTA, Tokyo, Japan), and the melanin index was calculated to evaluate the participants’ sun exposure during the intervention period^46^.

### Measurement of circulating level of factors

Blood was sampled by the same clinical nurse in the morning before the training intervention and within a week of the last exercise session. Participants were asked to fast for over 10 h before (water was permitted) blood sampling. The blood (20 mL) was divided into 2 tubes, processed into serum and plasma with ethylenediamine-*N*,*N*,*N*′,*N*′-tetraacetic acid, and stored at − 80 °C. Serum was used for the measurement of cytokines, and plasma was used to measure metabolites and to conduct the in vitro experiment with skin fibroblasts. The circulating factors measured in this study and references for the protocol about cytokine measurement are listed in Supplementary Table 1.

### Cell culturing with human blood plasma and circulating factors

NHDFs from a Caucasian woman (FC-0024, Lifeline Cell Technology, San Diego, CA, USA) in passage 8 were cultured on a 6-well plate with Dulbecco's Modified Eagle Medium (DMEM) containing 10% fetal bovine serum (FBS). After growing to 60–70% confluence, the cells were washed with phosphate-buffered saline (PBS) and treated with low-serum DMEM containing 0.1% FBS. After incubation at 37 °C, 5% CO_2_ for 48 h, the medium was replaced with DMEM containing 10% plasma from the participants; the plasma was previously frozen and stored at − 80 °C, and then heat inactivated at 56 °C for 30 min and filtered just before use in the experiments. Plasma was used for this in vitro experiment because it was reported to better mimic the circulating composition of the in vivo environment than serum^47^. At 6 h after replacing the medium with DMEM containing plasma, the cells were washed with PBS, and RNA was isolated. The confluent level of cells at the end of the experiment was about 80%. Six-hour incubation with plasma was determined to be appropriate for evaluating ECM-related gene expression level, because the expression level of tissue remodeling-related genes such as collagen and elastin in cultured skin fibroblasts, react and change after 6-h incubation with serum, and the profile of gene expression is maintained until after 24-h incubation^48^.

To examine the effect of circulating factors on skin fibroblasts, the cells in passage 8 were grown to 60–70% confluence on a 24-well plate with DMEM containing 10% FBS, washed with PBS, and treated with low-serum DMEM containing 0.1% FBS. After incubation for 48 h, the cells were treated with no-serum DMEM containing circulating factors at the approximate mean concentration measured in participant blood before the training intervention: the mean concentration of CCL28 was 150 pg/mL; *N*,*N*-dimethylglycine, 7 μM; CXCL4, 10 μg/mL; and CXCL8, 10 pg/mL. At 6 h after adding the no-serum DMEM containing circulating factors, the cells were washed with PBS, and RNA was isolated. The confluent level of cells at the end of the experiment was about 80%.

### Gene expression analysis

RNA isolated from fibroblasts was reversely transcribed to complementary DNA, and the expression of dermal ECM genes was analyzed with a quantitative polymerase chain reaction system (BioMark HD, Fluidigm, San Francisco, CA, USA). The threshold cycle (Ct) value of gene expression was calculated by software (Fluidigm Real-Time PCR Analysis, Fluidigm). Biological variation between samples was normalized with the Ct value of the glyceraldehyde 3-phosphate dehydrogenase gene (*GAPDH*) as a housekeeping gene. Experimental variation between culture plates was normalized on each plate with the Ct value of the control sample, which was treated with 10% FBS, and the relative value calculated by the ΔΔCt method was used for the analysis. To examine the effect of blood plasma or circulating factors on the dermis, the dermal ECM genes shown in Table 2 were quantified. The primers and probes of TaqMan^®^ Gene Expression Assay (Thermo Fisher Scientific, Waltham, MA, USA) shown in Supplementary Table 2 were used for the analysis.

### Statistical analysis

Statistical analyses of intragroup differences in changes in skin parameters, body composition, physical capacity, circulating levels of factors, and gene expression were performed with two-sided paired *t* tests (Figs. 2b,c, 3b, 4b, Table 1). The intergroup differences in the delta (Δ) of skin parameters, body composition, and physical capacities between the AT and RT groups were evaluated by analysis of co-variance with adjustment for the baseline value (Fig. 2d, Extended Data 2). Correlation analyses were performed by Pearson correlation analysis (Fig. 4c–f). Statistical analyses of the effect of blood factors on gene expression levels were performed with Dunnett’s test (Fig. 4g) and two-sided independent *t* tests (Table 3).

All statistical analyses were performed with JMP version 14 (SAS Institute, Cary, NC, USA). Probability values of less than 0.05 (p < 0.05) were considered significant, and p values of less than 0.10 were considered marginally significant.

## Supplementary Information

Supplementary Information.

## Data Availability

The data that support the findings of this study are openly available in https://figshare.com/ at http://doi.org/10.6084/m9.figshare.22216876.

## References

[1] Erickson KI (2011). Exercise training increases size of hippocampus and improves memory. Proc. Natl. Acad. Sci. USA.

[2] Horowitz AM (2020). Blood factors transfer beneficial effects of exercise on neurogenesis and cognition to the aged brain. Science (New York, NY).

[3] Maass A (2015). Vascular hippocampal plasticity after aerobic exercise in older adults. Mol. Psychiatry.

[4] Abreu P, Mendes SV, Ceccatto VM, Hirabara SM (2017). Satellite cell activation induced by aerobic muscle adaptation in response to endurance exercise in humans and rodents. Life Sci..

[5] Brett JO (2020). Exercise rejuvenates quiescent skeletal muscle stem cells in old mice through restoration of Cyclin D1. Nat. Metab..

[6] Crane JD (2015). Exercise-stimulated interleukin-15 is controlled by AMPK and regulates skin metabolism and aging. Aging Cell.

[7] Slominski A, Wortsman J (2000). Neuroendocrinology of the skin. Endocr. Rev..

[8] Slominski AT (2022). Neuroendocrine signaling in the skin with a special focus on the epidermal neuropeptides. Am. J. Physiol. Cell Physiol..

[9] McCabe MC (2020). Alterations in extracellular matrix composition during aging and photoaging of the skin. Matrix Biol. Plus..

[10] Passeron T, Krutmann J, Andersen ML, Katta R, Zouboulis CC (2020). Clinical and biological impact of the exposome on the skin. J. Eur. Acad. Dermatol. Venereol..

[11] Krutmann J, Bouloc A, Sore G, Bernard BA, Passeron T (2017). The skin aging exposome. J. Dermatol. Sci..

[12] Bocheva G, Slominski RM, Slominski AT (2019). Neuroendocrine aspects of skin aging. Int. J. Mol. Sci..

[13] Weinmüllner R (2020). Organotypic human skin culture models constructed with senescent fibroblasts show hallmarks of skin aging. NPJ Aging Mech. Dis..

[14] Branchet MC, Boisnic S, Frances C, Robert AM (1990). Skin thickness changes in normal aging skin. Gerontology.

[15] Petersen AM, Pedersen BK (2005). The anti-inflammatory effect of exercise. J. Appl. Physiol..

[16] Hoffmann C, Weigert C (2017). Skeletal muscle as an endocrine organ: The role of myokines in exercise adaptations. Cold Spring Harb Perspect. Med..

[17] Schnyder S, Handschin C (2015). Skeletal muscle as an endocrine organ: PGC-1α, myokines and exercise. Bone.

[18] Tsai CL, Pai MC, Ukropec J, Ukropcová B (2019). Distinctive effects of aerobic and resistance exercise modes on neurocognitive and biochemical changes in individuals with mild cognitive impairment. Curr. Alzheimer Res..

[19] Abd El-Kader SM, Al-Shreef FM, Al-Jiffri OH (2019). Impact of aerobic exercise versus resisted exercise on endothelial activation markers and inflammatory cytokines among elderly. Afr. Health Sci..

[20] Corsi A (2002). Phenotypic effects of biglycan deficiency are linked to collagen fibril abnormalities, are synergized by decorin deficiency, and mimic Ehlers-Danlos-like changes in bone and other connective tissues. J. Bone Miner. Res..

[21] Grgic J (2019). Does aerobic training promote the same skeletal muscle hypertrophy as resistance training? A systematic review and meta-analysis. Sports Med..

[22] Mann S, Beedie C, Jimenez A (2014). Differential effects of aerobic exercise, resistance training and combined exercise modalities on cholesterol and the lipid profile: Review, synthesis and recommendations. Sports Med..

[23] Phillips BE, Williams JP, Greenhaff PL, Smith K, Atherton PJ (2017). Physiological adaptations to resistance exercise as a function of age. JCI Insight.

[24] Ohshima H (2013). Use of Cutometer area parameters in evaluating age-related changes in the skin elasticity of the cheek. Skin Res. Technol..

[25] Takema Y, Yorimoto Y, Kawai M, Imokawa G (1994). Age-related changes in the elastic properties and thickness of human facial skin. Br. J. Dermatol..

[26] Gniadecka M (2001). Effects of ageing on dermal echogenicity. Skin Res. Technol..

[27] Sandby-Møller J, Wulf HC (2004). Ultrasonographic subepidermal low-echogenic band, dependence of age and body site. Skin Res. Technol..

[28] Tsukahara K (2000). Age-related alterations of echogenicity in Japanese skin. Dermatology.

[29] Ryu HS, Joo YH, Kim SO, Park KC, Youn SW (2008). Influence of age and regional differences on skin elasticity as measured by the Cutometer. Skin Res. Technol..

[30] Yang YL (2011). Influence of chondroitin sulfate and hyaluronic acid on structure, mechanical properties, and glioma invasion of collagen I gels. Biomaterials.

[31] Hsu S, Jamieson AM, Blackwell J (1994). Viscoelastic studies of extracellular matrix interactions in a model native collagen gel system. Biorheology.

[32] Hesselstrand R, Westergren-Thorsson G, Scheja A, Wildt M, Akesson A (2002). The association between changes in skin echogenicity and the fibroblast production of biglycan and versican in systemic sclerosis. Clin. Exp. Rheumatol..

[33] Lee DH, Oh JH, Chung JH (2016). Glycosaminoglycan and proteoglycan in skin aging. J. Dermatol. Sci..

[34] Bünemann E (2018). Chemokine ligand-receptor interactions critically regulate cutaneous wound healing. Eur. J. Med. Res..

[35] van Bon L (2014). Proteome-wide analysis and CXCL4 as a biomarker in systemic sclerosis. N Engl J Med..

[36] Buskermolen JK, Roffel S, Gibbs S (2017). Stimulation of oral fibroblast chemokine receptors identifies CCR3 and CCR4 as potential wound healing targets. J. Cell Physiol..

[37] Huen AC, Wells A (2012). The beginning of the end: CXCR3 signaling in late-stage wound healing. Adv. Wound Care (New Rochelle)..

[38] Van Raemdonck K, Van den Steen PE, Liekens S, Van Damme J, Struyf S (2015). CXCR3 ligands in disease and therapy. Cytokine Growth Factor Rev..

[39] Dolsen EA, Crosswell AD, Prather AA (2019). Links between stress, sleep, and inflammation: Are there sex differences?. Curr. Psychiatry Rep..

[40] Bergens O, Nilsson A, Papaioannou KG, Kadi F (2021). Sedentary patterns and systemic inflammation: Sex-specific links in older adults. Front. Physiol..

[41] Wanigatunga AA (2019). Longitudinal relationship between interleukin-6 and perceived fatigability among well-functioning adults in mid-to-late life. J. Gerontol. A Biol. Sci. Med. Sci..

[42] Kruger K (2018). Exercise training reverses inflammation and muscle wasting after tobacco smoke exposure. Am. J. Physiol. Regul. Integr. Comp. Physiol..

[43] Shimojo G (2019). Exercise activates vagal induction of dopamine and attenuates systemic inflammation. Brain. Behav. Immun..

[44] Yasuda J (2022). Relationship between protein intake and resistance training-induced muscle hypertrophy in middle-aged women: A pilot study. Nutrition (Burbank, Los Angeles County, Calif.).

[45] Yasuda J, Tomita T, Arimitsu T, Fujita S (2020). Evenly distributed protein intake over 3 meals augments resistance exercise-induced muscle hypertrophy in healthy young men. J. Nutr..

[46] Yun IS, Lee WJ, Rah DK, Kim YO, Park BY (2010). Skin color analysis using a spectrophotometer in Asians. Skin Res. Technol..

[47] Mirshafiee V, Kim R, Mahmoudi M, Kraft ML (2016). The importance of selecting a proper biological milieu for protein corona analysis in vitro: Human plasma versus human serum. Int. J. Biochem. Cell Biol..

[48] Iyer VR (1999). The transcriptional program in the response of human fibroblasts to serum. Science (New York, NY).

