# Supplementary material for: Resistance training rejuvenates aging skin by reducing circulating inflammatory factors and enhancing dermal extracellular matrices
Source: Sci Rep. 2023 Jun 23;13:10214. doi: 10.1038/s41598-023-37207-9 (PMC10290068; doi:10.1038/s41598-023-37207-9)
Supplement: Supplementary file 1 — Supplementary Information. [file 41598_2023_37207_MOESM1_ESM.pdf]

Resistance training rejuvenates aging skin by reducing circulating inflammatory factors and enhancing dermal extracellular matrices

Shu Nishikori[1,2], Jun Yasuda[1], Kao Murata[1], Junya Takegaki[1], Yasuko Harada[2], Yuki Shirai[2] and Satoshi Fujita[1]

[1] Faculty of Sports and Health Science, Ritsumeikan University, 1-1-1 Nojihigashi, Kusatsu, Japan
[2] Frontier Research Center, POLA Chemical Industries, Inc., 560 Kashio-cho, Totsuka-ku, Yokohama, Japan

Corresponding author:
*Satoshi Fujita[1]
Tel: +81-077-561-5229, E-mail: safujita@fc.ritsumei.ac.jp

Authors:
Shu Nishikori[1,2]
Tel: +81-090-2734-7983, E-mail: s-nishikori@pola.co.jp

Jun Yasuda[1]
E-mail: 55fhyanh@gmail.com

Kao Murata[1]
E-mail: chibimaru.ohana@gmail.com

Junya Takegaki[1]
E-mail: takegakij@gmail.com

Yasuko Harada[2]
E-mail: ya-harada@pola.co.jp

Yuki Shirai[2]
E-mail: y-shirai@pola.co.jp

Extended Data 1. Characteristics of participants in the aerobic training and resistance training groups

	All (n = 56)	AT (n = 26)	RT (n = 30)	p-value
Age, y	48.5 ± 5.2	49.5 ± 5.1	47.7 ± 5.1	0.19
Regular menstruation: Yes, n (%)	27 (48)	10 (38)	17 (57)	0.17[a]
Dietary intake				
Energy, kcal/day	1805.4 ± 319.2	1837.1 ± 241.8	1774 ± 381.5	0.48
Protein, g/day	73.2 ± 13.4	73.6 ± 10.8	72.9 ± 15.8	0.85
Fat, g/day	68.9 ± 16.3	71.1 ± 13.6	66.8 ± 18.6	0.34
Carbohydrate, g/day	214.0 ± 40.9	217.4 ± 33.1	210.8 ± 47.7	0.56
Skin aging parameters				
Skin elasticity, Ur/Uf	0.32 ± 0.05	0.32 ± 0.05	0.32 ± 0.05	0.60
Dermal thickness, mm	1.74 ± 0.16	1.71 ± 0.19	1.77 ± 0.14	0.18
Upper dermal LEP, %	27.0 ± 13.9	27.9 ± 15.4	26.3 ± 12.7	0.69
Body composition				
Height, m	1.61 ± 0.05	1.60 ± 0.06	1.61 ± 0.05	0.77
Body weight, kg	54.4 ± 6.1	54.2 ± 6.5	54.6 ± 5.8	0.84
BMI, kg/m^2	21.1 ± 1.6	21.0 ± 1.7	21.1 ± 1.5	0.94
AppLTM, kg	15.4 ± 1.8	15.4 ± 2.0	15.4 ± 1.7	0.87
TotalLTM, kg	36.5 ± 3.7	36.7 ± 4.1	36.4 ± 3.4	0.8
Body fat, kg	15.6 ± 4.1	15.2 ± 3.9	15.9 ± 4.4	0.57
BMC, kg	2.28 ± 0.30	2.25 ± 0.32	1.31 ± 0.28	0.47
Physical capacity				
VO_{2peak}, mL/kg/min	28.0 ± 4.8	27.1 ± 0.9	28.7 ± 0.9	0.20
1-RM, kg				
Chest press	11.5 ± 3.3	11.2 ± 3.2	11.8 ± 3.3	0.19
Shoulder press	15.7 ± 3.7	14.9 ± 3.8	16.3 ± 3.6	0.49
Arm curl	15.2 ± 3.4	15.0 ± 3.9	15.4 ± 3	0.30
Rowing	22.1 ± 4.0	21.7 ± 4.9	22.5 ± 3.1	0.30
Leg curl	43.4 ± 8.9	42.0 ± 10.1	44.6 ± 7.8	0.44
Leg extension	68.2 ± 17.8	65.5 ± 18.9	70.5 ± 16.7	0.15

Values show the means ± standard deviations (SDs). Statistical analyses were performed with two-sided independent t tests and [a]chi-squared test to compare the aerobic training and resistance training groups.
AppLTM, appendicular lean soft tissue mass; AT, aerobic training; BMC, bone mineral content; BMI, body mass index; LEP, low echogenic pixel; RT, resistance training; TotalLTM, total lean soft tissue mass; VO_{2peak}, peak oxygen uptake; 1-RM, one-repetition maximum

Extended Data 2. Differences in the effect of aerobic training and resistance training on skin aging parameters, body composition, and physical capacity

	ANCOVA 1[a]			ANCOVA 2[b]			ANCOVA 3[c]		
	AT (n = 26)	RT (n = 30)	p value	AT (n = 26)	RT (n = 30)	p value	AT (n = 26)	RT (n = 30)	p value
Skin aging parameters									
ΔSkin elasticity, Ur/Uf	+0.045 ± 0.008	+0.056 ± 0.007	0.29	+0.042 ± 0.007	+0.058 ± 0.007	0.13	+0.043 ± 0.008	+0.057 ± 0.007	0.16
ΔDermal thickness, mm	-0.014 ± 0.023	+0.040 ± 0.021	< 0.10	-0.015 ± 0.023	+0.041 ± 0.022	< 0.10	-0.014 ± 0.023	+0.041 ± 0.022	< 0.10
ΔUpper dermal LEPs, %	-12.1 ± 2.4	-11.0 ± 2.2	0.98	-12.1 ± 2.4	-11.0 ± 2.2	0.98	-12.1 ± 2.4	-11.0 ± 2.2	0.88
Body composition									
ΔBody weight, kg	-0.73 ± 0.26	-0.067 ± 0.25	< 0.10	-0.75 ± 0.27	-0.046 ± 0.25	< 0.10			
ΔBMI, kg/m^2	-0.25 ± 0.10	-0.0091 ± 0.0899	< 0.10	-0.26 ± 0.10	+0.0019 ± 0.091	< 0.10			
ΔAppLTM, kg	+0.0062 ± 0.0733	+0.24 ± 0.07	< 0.05	+0.021 ± 0.073	+0.23 ± 0.07	< 0.05			
ΔTotalLTM, kg	-0.054 ± 0.148	+0.43 ± 0.14	< 0.05	-0.041 ± 0.150	+0.42 ± 0.14	< 0.05			
ΔBody fat, kg	-0.36 ± 0.24	-0.21 ± 0.23	0.67	-0.40 ± 0.24	-0.17 ± 0.22	0.49			
ΔBMC, kg	-0.0085 ± 0.0069	-0.0070 ± 0.0064	0.88	-0.0079 ± 0.0070	-0.0076 ± 0.0065	0.97			
Physical capacity									
ΔVO_{2peak}, mL/kg/min	+1.01 ± 0.67	+0.91 ± 0.62	0.91	+1.06 ± 0.68	+0.87 ± 0.63	0.84			
Δ1-RM (kg)									
ΔChest press	+0.46 ± 0.32	+5.9 ± 0.3	< 0.001	+0.49 ± 0.32	+5.8 ± 0.3	< 0.001			
ΔShoulder press	+0.71 ± 0.50	+7.5 ± 0.5	< 0.001	+0.73 ± 0.51	+7.5 ± 0.5	< 0.001			
ΔArm curl	+0.54 ± 0.53	+6.4 ± 0.5	< 0.001	+0.62 ± 0.53	+6.3 ± 0.5	< 0.001			
ΔRowing	-0.94 ± 0.57	+5.4 ± 0.5	< 0.001	-0.84 ± 0.57	+5.3 ± 0.5	< 0.001			
ΔLeg curl	+0.48 ± 1.28	+12.4 ± 1.2	< 0.001	+0.74 ± 1.24	+12.2 ± 1.2	< 0.001			
ΔLeg extension	+2.6 ± 1.6	+16.0 ± 1.5	< 0.001	+2.7 ± 1.7	+15.9 ± 1.6	< 0.001			

Values show the means of delta (Δ) calculated by subtracting the baseline value measured before the training intervention from the value measured after the training intervention, ± standard errors (SEs). Statistical analyses were performed with three types of analysis of co-variance by adjusting for [a]the baseline value measured before training intervention, [b]the baseline value measured before training intervention and menstruation regularity (regular or irregular), and [c]the baseline value measured before training intervention, menstruation regularity (regular/irregular or after menopause), and Δmelanin index as a measure of exposure to ultraviolet rays during the intervention period.

ANCOVA, analysis of co-variance; AppLTM, appendicular lean soft tissue mass; AT, aerobic training; BMC, bone mineral content; BMI, body mass index; LEP, low echogenic pixel; RT, resistance training; TotalLTM, total lean soft tissue mass; VO_2peak, peak oxygen uptake; 1-RM, one-repetition maximum

Extended Data 3. Blood factors that decreased significantly after both aerobic training and resistance training

Blood factor	Unit	All (n = 56)			AT (n = 26)			RT (n = 30)			Change (Δ)		
		Before	After	p value	Before	After	p value	Before	After	p value	AT	RT	p value[a]
Total protein	g/dL	7.03 ± 0.07	6.85 ± 0.07	< 0.01	6.84 ± 0.11	6.67 ± 0.13	< 0.10	7.19 ± 0.08	7.00 ± 0.07	< 0.05	-0.17 ± 0.08	-0.19 ± 0.08	0.87
Albumin	g/dL	4.31 ± 0.04	4.18 ± 0.04	< 0.01	4.22 ± 0.06	4.10 ± 0.07	< 0.10	4.39 ± 0.05	4.25 ± 0.05	< 0.01	-0.12 ± 0.05	-0.14 ± 0.05	0.87
Insulin	µU/mL	5.66 ± 0.37	4.35 ± 0.25	< 0.0001	6.25 ± 0.74	4.49 ± 0.50	< 0.01	5.15 ± 0.25	4.24 ± 0.22	< 0.01	-1.93 ± 0.27	-0.91 ± 0.27	< 0.10
Eotaxin	pg/mL	23.92 ± 1.07	21.64 ± 1.01	< 0.01	21.71 ± 1.16	19.80 ± 1.07	< 0.01	25.84 ± 1.67	23.23 ± 1.60	< 0.05	-1.91 ± 1.24	-2.61 ± 1.24	0.64
IL-18	pg/mL	47.22 ± 4.22	40.08 ± 2.87	< 0.05	38.75 ± 1.59	34.51 ± 1.35	< 0.01	54.56 ± 7.55	44.91 ± 5.10	< 0.10	-4.24 ± 5.20	-9.65 ± 5.20	0.35
LIF	FI[b]	1.99 ± 0.82	0.99 ± 0.77	< 0.001	3.79 ± 1.43	2.25 ± 1.17	< 0.01	0.42 ± 0.81	-0.10 ± 1.01	< 0.05	-1.54 ± 0.50	-0.52 ± 0.58	0.19
M-CSF	pg/mL	13.74 ± 0.75	11.59 ± 0.51	< 0.001	13.23 ± 1.08	10.92 ± 0.76	< 0.01	14.19 ± 1.04	12.16 ± 0.67	< 0.05	-2.31 ± 0.94	-2.03 ± 0.94	0.82
I-309/CCL1	pg/mL	27.09 ± 0.49	25.71 ± 0.56	< 0.01	26.92 ± 0.68	25.80 ± 0.85	< 0.05	27.23 ± 0.71	25.62 ± 0.77	< 0.05	-1.12 ± 0.62	-1.61 ± 0.62	0.56
IGF-I	ng/mL	61.52 ± 2.42	54.25 ± 2.46	< 0.0001	62.35 ± 4.09	54.34 ± 3.66	< 0.01	60.81 ± 2.85	54.17 ± 3.39	< 0.01	-8.00 ± 1.96	-6.64 ± 1.96	0.66
ACTH	pg/mL	12.54 ± 0.90	9.88 ± 0.69	< 0.001	10.54 ± 1.08	7.82 ± 0.67	< 0.01	14.34 ± 1.33	11.72 ± 1.06	< 0.05	-2.72 ± 1.20	-2.53 ± 1.20	0.90
afp	µIU/mL	4.06 ± 0.43	3.11 ± 0.31	< 0.01	3.46 ± 0.43	2.63 ± 0.38	< 0.10	4.58 ± 0.71	3.53 ± 0.48	< 0.05	-0.83 ± 0.40	-1.17 ± 0.40	0.56
EGF	pg/mL	186.07 ± 10.62	107.26 ± 10.44	< 0.0001	179.68 ± 15.45	113.54 ± 17.14	< 0.01	191.62 ± 14.80	101.82 ± 12.80	< 0.0001	-66.14 ± 17.86	-89.80 ± 17.86	0.38
FLRG	ng/mL	17.61 ± 0.59	15.66 ± 0.47	< 0.0001	17.46 ± 0.83	15.61 ± 0.65	< 0.01	17.74 ± 0.84	15.71 ± 0.69	< 0.01	-1.85 ± 0.57	-2.03 ± 0.57	0.83
NT-Pro-ANP	ng/mL	6.44 ± 0.36	5.58 ± 0.35	< 0.001	5.70 ± 0.50	4.97 ± 0.53	< 0.05	7.08 ± 0.50	6.11 ± 0.45	< 0.01	-0.73 ± 0.28	-0.97 ± 0.28	0.60
Prolactin	ng/mL	26.2 ± 4.72	18.99 ± 3.54	< 0.001	21.46 ± 3.04	14.79 ± 2.14	< 0.01	30.32 ± 8.40	22.63 ± 6.33	< 0.05	-6.67 ± 2.93	-7.69 ± 2.93	0.78
Chemerin	ng/mL	5.40 ± 0.21	5.07 ± 0.17	< 0.01	5.70 ± 0.30	5.31 ± 0.22	< 0.05	5.14 ± 0.30	4.87 ± 0.25	< 0.10	-0.39 ± 0.14	-0.28 ± 0.14	0.61
CD40 Ligand/TNFSF5	ng/mL	8.98 ± 0.32	6.81 ± 0.33	< 0.0001	8.38 ± 0.46	6.63 ± 0.46	< 0.001	9.49 ± 0.42	6.96 ± 0.47	< 0.001	-1.76 ± 0.60	-2.53 ± 0.60	0.30
Flt-3 Ligand	pg/mL	127.94 ± 5.63	116.72 ± 5.32	< 0.0001	127.83 ± 9.68	117.81 ± 9.11	< 0.01	128.03 ± 6.50	115.78 ± 6.17	< 0.0001	-10.01 ± 1.99	-12.25 ± 1.99	0.55
VEGFR2/KDR/Flk-1	ng/mL	10.39 ± 0.38	9.93 ± 0.37	< 0.001	10.33 ± 0.57	9.89 ± 0.56	< 0.05	10.44 ± 0.52	9.96 ± 0.51	< 0.05	-0.43 ± 0.19	-0.48 ± 0.19	0.85
Syndecan-1/CD138	ng/mL	2.66 ± 0.07	2.44 ± 0.07	< 0.0001	2.63 ± 0.10	2.37 ± 0.10	< 0.0001	2.69 ± 0.10	2.50 ± 0.09	< 0.001	-0.25 ± 0.04	-0.19 ± 0.04	0.32
Dkk-1	ng/mL	3.33 ± 0.14	3.08 ± 0.14	< 0.01	3.03 ± 0.13	2.87 ± 0.13	< 0.05	3.59 ± 0.23	3.26 ± 0.24	< 0.05	-0.16 ± 0.14	-0.33 ± 0.14	0.32
Cathepsin S	ng/mL	3.04 ± 0.17	2.81 ± 0.16	< 0.01	3.05 ± 0.29	2.77 ± 0.27	< 0.10	3.04 ± 0.21	2.84 ± 0.18	< 0.01	-0.27 ± 0.06	-0.20 ± 0.06	0.64
Osteoprotegerin	pg/mL	763.14 ± 20.66	723.33 ± 20.15	< 0.0001	737.09 ± 22.75	690.81 ± 22.13	< 0.001	785.71 ± 32.97	751.52 ± 31.81	< 0.01	-46.28 ± 10.76	-34.19 ± 10.76	0.46
Follistatin-like 1/FSTL1	ng/mL	10.65 ± 0.23	10.18 ± 0.25	< 0.0001	10.48 ± 0.38	9.80 ± 0.41	< 0.001	10.80 ± 0.29	10.5 ± 0.31	< 0.05	-0.67 ± 0.11	-0.30 ± 0.11	< 0.10
IL-1 R1	ng/mL	1.45 ± 0.03	1.40 ± 0.03	< 0.01	1.40 ± 0.03	1.34 ± 0.03	< 0.05	1.49 ± 0.04	1.46 ± 0.04	< 0.10	-0.06 ± 0.01	-0.03 ± 0.01	0.25
HB-EGF	pg/mL	230.57 ± 9.91	204.46 ± 9.67	< 0.01	216.11 ± 11.42	199.12 ± 11.53	< 0.05	243.10 ± 15.45	209.09 ± 15.17	< 0.05	-16.99 ± 12.93	-34.01 ± 12.93	0.28
SPARC	µg/mL	1.48 ± 0.04	1.35 ± 0.05	< 0.01	1.43 ± 0.06	1.35 ± 0.06	< 0.05	1.52 ± 0.06	1.36 ± 0.07	< 0.05	-0.08 ± 0.07	-0.16 ± 0.07	0.38
HMW Adiponectin	µg/mL	7.91 ± 0.60	6.36 ± 0.48	< 0.0001	8.46 ± 0.89	7.35 ± 0.79	< 0.01	7.42 ± 0.81	5.50 ± 0.53	< 0.0001	-1.11 ± 0.37	-1.92 ± 0.37	0.14
PDGF-AA	ng/mL	4.35 ± 0.14	3.98 ± 0.14	< 0.01	4.23 ± 0.20	3.96 ± 0.18	< 0.05	4.45 ± 0.21	4.00 ± 0.22	< 0.10	-0.27 ± 0.23	-0.45 ± 0.23	0.50
Angiopoietin-1	ng/mL	59.99 ± 2.08	54.9 ± 2.10	< 0.01	59.08 ± 3.01	55.76 ± 2.76	< 0.10	60.78 ± 2.92	54.16 ± 3.14	< 0.05	-3.31 ± 3.04	-6.62 ± 3.04	0.37
TIMP-1	ng/mL	120.28 ± 2.43	110.87 ± 2.84	< 0.0001	113.14 ± 3.5	108.35 ± 3.82	< 0.05	126.46 ± 2.99	113.06 ± 4.15	< 0.001	-4.80 ± 3.21	-13.4 ± 3.21	< 0.05
CD14	µg/mL	1.42 ± 0.06	1.28 ± 0.04	< 0.01	1.45 ± 0.09	1.28 ± 0.04	< 0.10	1.39 ± 0.07	1.28 ± 0.07	< 0.05	-0.17 ± 0.04	-0.11 ± 0.04	0.53
Serpin E1/PAI-1	ng/mL	95.21 ± 3.20	85.45 ± 3.31	< 0.001	90.31 ± 4.74	82.78 ± 3.55	< 0.05	99.46 ± 4.27	87.77 ± 5.38	< 0.05	-7.53 ± 4.28	-11.69 ± 4.28	0.43
Adiponectin/Acrp30	µg/mL	6.71 ± 0.30	5.94 ± 0.25	< 0.0001	6.83 ± 0.44	6.24 ± 0.42	< 0.01	6.61 ± 0.41	5.68 ± 0.30	< 0.001	-0.59 ± 0.21	-0.93 ± 0.21	0.26
CXCL7/NAP-2	µg/mL	2.88 ± 0.06	2.65 ± 0.07	< 0.01	2.77 ± 0.08	2.66 ± 0.08	< 0.10	2.97 ± 0.10	2.64 ± 0.12	< 0.01	-0.11 ± 0.11	-0.33 ± 0.11	< 0.10
Serpin A7/TBG	µg/mL	10.80 ± 0.22	10.26 ± 0.21	< 0.001	10.82 ± 0.33	10.36 ± 0.34	< 0.05	10.79 ± 0.30	10.18 ± 0.27	< 0.01	-0.46 ± 0.23	-0.60 ± 0.23	0.65
Angiogenin	ng/mL	293.74 ± 9.35	263.78 ± 7.66	< 0.0001	301.08 ± 13.01	272.16 ± 10.42	< 0.0001	287.39 ± 13.42	256.51 ± 11.08	< 0.001	-28.92 ± 7.86	-30.87 ± 7.86	0.85
Properdin	µg/mL	17.71 ± 0.39	16.68 ± 0.34	< 0.001	17.28 ± 0.49	16.47 ± 0.53	< 0.05	18.08 ± 0.58	16.87 ± 0.45	< 0.01	-0.82 ± 0.40	-1.21 ± 0.40	0.45
Serpin A10/ZPI	µg/mL	3.72 ± 0.11	3.52 ± 0.10	< 0.01	3.77 ± 0.20	3.56 ± 0.16	< 0.10	3.68 ± 0.12	3.48 ± 0.12	< 0.05	-0.21 ± 0.08	-0.20 ± 0.08	0.94
Serpin C1	µg/mL	936.71 ± 54.92	647.98 ± 46.04	< 0.0001	878.34 ± 81.49	650.24 ± 73.24	< 0.05	985.35 ± 74.40	646.02 ± 59.13	< 0.001	-194.32 ± 85.68	-339.33 ± 85.68	0.25
3-Aminobutyric acid	µM	0.43 ± 0.01	0.37 ± 0.01	< 0.001	0.43 ± 0.02	0.37 ± 0.02	< 0.05	0.43 ± 0.01	0.37 ± 0.01	< 0.01	-0.30 ± 0.08	-0.18 ± 0.08	0.26
Lactic acid	µM	1256.73 ± 92.18	882.32 ± 52.84	< 0.001	1326.93 ± 172.08	765.78 ± 56.98	< 0.01	1195.9 ± 87.65	983.32 ± 81.87	< 0.10	-395.74 ± 78.89	-258.21 ± 78.89	0.43
S-Methylcysteine	µM	5.91 ± 0.25	4.93 ± 0.25	< 0.01	5.83 ± 0.36	4.77 ± 0.39	< 0.05	5.97 ± 0.36	5.07 ± 0.33	< 0.10	-1.15 ± 0.46	-0.94 ± 0.46	0.76

Values show the means ± standard errors (SEs). The change value was calculated as delta (Δ) by subtracting the baseline value measured before the training intervention from the value measured after the training intervention. Statistical analyses were performed with two-sided paired t tests for the intragroup difference, [a]two-sided independent t tests for the intergroup difference, and [b]Wilcoxon signed-rank sum tests for the intragroup difference in the FI value.
AT, aerobic training; FI, fluorescence intensity; IL, interleukin; U, international unit of insulin; RT, resistance training

Extended Data 4. Blood factors that increased significantly after both aerobic training and resistance training

Blood factor	Unit	All (n= 56)			AT (n = 26)			RT (n = 30)			Change (Δ)		
		Before	After	*p value*	Before	After	*p value*	Before	After	*p value*	AT	RT	*p value* [a]
IL-26	FI[b]	4.97 ± 0.69	7.25 ± 0.87	< 0.001	5.65 ± 1.36	8.92 ± 1.34	< 0.01	4.38 ± 0.54	5.80 ± 1.10	< 0.05	+3.27 ± 1.00	+1.42 ± 1.00	0.20
Osteocalcin	ng/mL	3.79 ± 0.27	4.46 ± 0.30	< 0.0001	3.84 ± 0.50	4.29 ± 0.53	< 0.01	3.76 ± 0.27	4.62 ± 0.33	< 0.001	+0.60 ± 0.30	+0.71 ± 0.30	0.78
Osteopontin	ng/mL	15.51 ± 0.75	18.63 ± 0.91	< 0.001	16.21 ± 1.36	19.76 ± 1.46	< 0.01	14.92 ± 0.76	17.66 ± 1.13	< 0.01	+3.55 ± 0.99	+2.74 ± 0.99	0.61
Ghrelin	FI[b]	144.63 ± 43.85	356.91 ± 63.44	< 0.0001	194.22 ± 86.20	340.72 ± 98.53	< 0.05	101.66 ± 33.45	370.93 ± 83.56	< 0.001	+146.50 ± 80.54	+269.28 ± 80.54	0.29
ANGPTL6	ng/mL	4.67 ± 0.26	5.28 ± 0.30	< 0.01	4.38 ± 0.33	4.85 ± 0.42	< 0.05	4.91 ± 0.40	5.66 ± 0.43	< 0.05	+0.46 ± 0.30	+0.76 ± 0.30	0.44
Angiopoietin-2	ng/mL	1.81 ± 0.09	2.10 ± 0.12	< 0.0001	1.79 ± 0.15	2.13 ± 0.21	< 0.01	1.83 ± 0.12	2.08 ± 0.13	< 0.001	+0.34 ± 0.06	+0.24 ± 0.06	0.44
Synuclein-α	pg/mL	490.64 ± 15.82	689.89 ± 23.82	< 0.0001	486.17 ± 16.53	679.71 ± 43.88	< 0.001	494.50 ± 26.09	698.72 ± 23.77	< 0.0001	+193.54 ± 31.45	+204.21 ± 31.45	0.84
TGF-α	FI[b]	20.94 ± 0.38	22.25 ± 0.48	< 0.01	21.02 ± 0.60	22.19 ± 0.75	< 0.05	20.87 ± 0.48	22.30 ± 0.63	< 0.05	+1.17 ± 0.71	+1.43 ± 0.71	0.77
CCL14/HCC-1/HCC-3	ng/mL	42.03 ± 1.07	44.81 ± 0.97	< 0.0001	39.47 ± 1.62	42.88 ± 1.47	< 0.001	44.24 ± 1.31	46.49 ± 1.23	< 0.01	+3.41 ± 0.81	+2.25 ± 0.81	0.34
Glu	μM	43.10 ± 1.60	51.57 ± 2.31	< 0.001	41.44 ± 1.84	52.57 ± 3.80	< 0.01	44.55 ± 2.52	50.70 ± 2.82	< 0.05	+10.81 ± 2.49	+5.60 ± 2.49	0.25
Homocitrulline	μM	0.16 ± 0.01	0.19 ± 0.01	< 0.001	0.16 ± 0.01	0.18 ± 0.02	< 0.05	0.16 ± 0.01	0.19 ± 0.02	< 0.01	+0.26 ± 0.07	+0.11 ± 0.07	0.22
SDMA	μM	0.34 ± 0.01	0.38 ± 0.01	< 0.0001	0.34 ± 0.01	0.38 ± 0.01	< 0.01	0.34 ± 0.01	0.38 ± 0.01	< 0.01	+0.20 ± 0.09	+0.35 ± 0.09	0.26
Urea	μM	2100.02 ± 54.71	2280.97 ± 55.84	< 0.001	2094.63 ± 84.06	2247.97 ± 84.58	< 0.10	2104.70 ± 72.90	2309.57 ± 75.04	< 0.01	+82.73 ± 68.78	+172.78 ± 68.78	0.35

Values show the means ± standard errors (SEs). The change value was calculated by subtracting the baseline value measured before the training intervention from the value measured after the training intervention. Statistical analyses were performed with two-sided paired t tests for the intragroup difference, [a]two-sided independent t tests for the intergroup difference, and [b]Wilcoxon signed-rank sum tests for the intragroup difference in the FI value.

AT, aerobic training; FI, fluorescence intensity; RT, resistance training

Extended Data 5. Blood factors that decreased significantly only after aerobic training

Blood factor	Unit	All (n = 56)			AT (n = 26)			RT (n = 30)			Change (Δ)		
		Before	After	*p value*	Before	After	*p value*	Before	After	*p value*	AT	RT	*p value* [a]
HGF	pg/mL	246.41 ± 12.06	227.63 ± 9.45	< 0.05	242.67 ± 23.22	218.04 ± 16.8	< 0.05	249.65 ± 10.55	235.94 ± 10.00	0.22	-24.63 ± 10.88	-13.71 ± 10.88	0.47
IFN-γ	pg/mL	15.07 ± 4.65	12.57 ± 3.62	< 0.10	21.01 ± 9.93	15.08 ± 7.18	< 0.05	9.94 ± 1.09	10.07 ± 1.15	0.81	-5.12 ± 1.26	-0.89 ± 1.26	0.14
IL-2Ra	pg/mL	46.22 ± 2.06	43.72 ± 1.67	0.16	48.09 ± 4.05	42.95 ± 2.64	< 0.10	44.59 ± 1.63	44.38 ± 2.15	0.92	-5.14 ± 1.97	-0.21 ± 1.97	0.16
IL-4	pg/mL	2.28 ± 0.11	2.18 ± 0.11	0.20	2.49 ± 0.20	2.27 ± 0.19	< 0.05	2.11 ± 0.11	2.11 ± 0.12	0.98	-0.23 ± 0.12	0.00 ± 0.12	0.15
IL-12(p40)	pg/mL	63.12 ± 7.64	52.32 ± 7.00	< 0.10	72.29 ± 15.12	46.38 ± 11.07	< 0.01	55.84 ± 6.64	57.62 ± 8.88	0.92	-19.35 ± 9.47	-0.20 ± 9.47	0.14
IL-13	pg/mL	2.26 ± 0.26	1.98 ± 0.22	< 0.10	2.75 ± 0.54	2.22 ± 0.41	< 0.05	1.85 ± 0.16	1.79 ± 0.20	0.72	-0.59 ± 0.16	-0.06 ± 0.16	< 0.10
SCF	pg/mL	65.95 ± 2.25	63.74 ± 2.35	0.25	64.86 ± 3.38	60.31 ± 3.31	< 0.10	66.90 ± 3.06	66.72 ± 3.27	0.95	-4.56 ± 2.98	-0.18 ± 2.98	0.26
SCGF-b	ng/mL	138.86 ± 4.27	132.28 ± 4.27	< 0.01	132.39 ± 5.52	121.58 ± 5.14	< 0.001	144.46 ± 6.27	141.55 ± 6.20	0.33	-10.82 ± 2.93	-2.91 ± 2.93	< 0.05
TRAIL	pg/mL	47.61 ± 1.65	44.39 ± 1.15	< 0.05	49.63 ± 3.07	43.07 ± 1.48	< 0.05	45.87 ± 1.52	45.55 ± 1.71	0.78	-6.56 ± 1.16	-0.32 ± 1.16	< 0.05
Eotaxin-3/CCL26	pg/mL	11.33 ± 0.67	10.32 ± 0.68	< 0.01	12.08 ± 1.32	10.77 ± 1.35	< 0.01	10.69 ± 0.51	9.92 ± 0.53	0.11	-1.32 ± 0.47	-0.76 ± 0.47	0.37
Gro-β/CXCL2	pg/mL	130.49 ± 8.22	117.92 ± 7.71	< 0.05	123.02 ± 12.00	104.54 ± 8.42	< 0.05	136.96 ± 11.35	129.52 ± 12.14	0.30	-18.48 ± 7.02	-7.43 ± 7.02	0.29
TARC/CCL17	pg/mL	36.33 ± 2.36	30.59 ± 2.74	< 0.05	35.35 ± 3.56	28.67 ± 2.98	< 0.05	37.18 ± 3.18	32.19 ± 4.40	0.21	-7.78 ± 3.90	-4.99 ± 3.90	0.58
TECK/CCL25	pg/mL	290.71 ± 8.62	269.59 ± 8.42	< 0.01	295.98 ± 12.83	268.11 ± 9.00	< 0.05	286.15 ± 11.78	270.88 ± 13.8	0.14	-27.87 ± 10.01	-15.27 ± 10.01	0.39
Amylin	FI[b]	3.17 ± 0.62	2.30 ± 0.62	0.10	4.10 ± 0.97	2.52 ± 0.71	< 0.10	2.36 ± 0.79	2.11 ± 0.98	0.62	-1.59 ± 0.67	-0.25 ± 0.67	0.22
Cortisol	ng/mL	78.93 ± 2.51	72.53 ± 2.41	< 0.01	78.98 ± 4.20	69.26 ± 3.28	< 0.05	78.88 ± 3.03	75.37 ± 3.46	0.20	-9.72 ± 2.69	-3.51 ± 2.69	0.16
T4	ng/mL	58.67 ± 2.62	53.51 ± 1.51	< 0.05	62.94 ± 5.25	55.16 ± 2.77	< 0.10	54.98 ± 1.64	52.08 ± 1.47	0.18	-7.78 ± 2.11	-2.9 ± 2.11	0.25
C-Peptide	pg/mL	735.96 ± 36.42	686.26 ± 30.23	< 0.10	752.58 ± 65.44	648.87 ± 45.32	< 0.05	721.55 ± 38.48	718.66 ± 40.28	0.92	-103.71 ± 28.72	-2.89 ± 28.72	< 0.10
IL-4Rα	pg/mL	29.94 ± 0.77	29.56 ± 0.72	0.24	30.36 ± 1.03	29.10 ± 0.81	< 0.05	29.58 ± 1.15	29.96 ± 1.15	0.25	-1.26 ± 0.33	0.39 ± 0.33	< 0.01
AgRP/ART	pg/mL	41.54 ± 3.63	40.04 ± 3.29	0.12	46.69 ± 7.42	43.32 ± 6.87	< 0.05	37.07 ± 2.01	37.21 ± 1.60	0.89	-3.37 ± 0.99	0.13 ± 0.99	< 0.10
PDGF-CC	ng/mL	3.82 ± 0.17	3.47 ± 0.17	< 0.01	3.72 ± 0.26	3.33 ± 0.20	< 0.05	3.92 ± 0.24	3.59 ± 0.26	0.10	-0.39 ± 0.20	-0.33 ± 0.20	0.81
GDF-15	pg/mL	434.03 ± 21.85	406.36 ± 16.12	< 0.10	482.08 ± 39.52	423.09 ± 26.85	< 0.10	392.39 ± 19.85	391.86 ± 19.11	0.95	-58.99 ± 9.19	-0.53 ± 9.19	< 0.10
Growth hormone	ng/mL	4.84 ± 0.61	3.55 ± 0.33	< 0.05	4.74 ± 0.85	2.98 ± 0.48	< 0.10	4.93 ± 0.87	4.05 ± 0.45	0.26	-1.76 ± 0.77	-0.88 ± 0.77	0.46
Renin	pg/mL	568.57 ± 149.94	548.04 ± 138.84	0.67	700.68 ± 293.74	538.13 ± 230.40	< 0.05	454.07 ± 118.99	556.64 ± 169.03	< 0.10	-162.56 ± 60.06	+102.57 ± 60.06	< 0.01
XCL1/Lymphotactin	pg/mL	42.76 ± 2.88	40.41 ± 2.97	0.22	42.98 ± 2.47	34.39 ± 2.29	< 0.01	42.58 ± 4.98	45.63 ± 5.03	0.17	-8.59 ± 2.20	+3.05 ± 2.20	< 0.01
Osteoactivin/GPNMB	ng/mL	16.43 ± 0.33	15.95 ± 0.34	< 0.05	15.27 ± 0.49	14.6 ± 0.45	< 0.05	17.43 ± 0.38	17.12 ± 0.39	0.25	-0.67 ± 0.26	-0.31 ± 0.26	0.34
TNF R2/TNFRSF1B	ng/mL	1.65 ± 0.08	1.54 ± 0.06	< 0.05	1.78 ± 0.14	1.58 ± 0.09	< 0.10	1.55 ± 0.09	1.51 ± 0.08	0.25	-0.19 ± 0.03	-0.04 ± 0.03	0.16
TNF R1/TNFRSF1A	pg/mL	947.99 ± 29.66	908.59 ± 24.85	< 0.05	942.71 ± 52.65	851.73 ± 35.20	< 0.01	952.56 ± 32.28	957.87 ± 32.87	0.70	-90.98 ± 13.44	+5.31 ± 13.44	< 0.01
Ferritin	ng/mL	40.66 ± 5.02	33.83 ± 3.48	< 0.05	47.93 ± 8.40	37.30 ± 5.02	< 0.10	34.35 ± 5.79	30.81 ± 4.83	0.14	-10.63 ± 2.36	-3.54 ± 2.36	0.20
2-Oxoisovaleric acid	μM	13.14 ± 0.31	12.28 ± 0.35	< 0.10	13.48 ± 0.52	12.08 ± 0.47	< 0.05	12.85 ± 0.36	12.46 ± 0.52	0.54	-1.4 ± 0.62	-0.37 ± 0.62	0.24
Ala	μM	492.54 ± 13.14	472.47 ± 12.81	0.16	492.85 ± 18.93	450.64 ± 15.79	< 0.05	492.27 ± 18.52	491.39 ± 19.19	0.97	-22.14 ± 19.19	4.23 ± 19.19	0.31
Argininosuccinic acid	μM	0.14 ± 0.00	0.14 ± 0.01	0.82	0.14 ± 0.01	0.13 ± 0.01	< 0.10	0.14 ± 0.01	0.15 ± 0.01	0.34	-0.25 ± 0.08	+0.16 ± 0.08	< 0.01
Butyrylcarnitine	μM	0.18 ± 0.03	0.16 ± 0.02	< 0.10	0.19 ± 0.04	0.17 ± 0.04	< 0.05	0.18 ± 0.04	0.15 ± 0.01	0.23	-0.14 ± 0.09	-0.01 ± 0.09	0.38
cis-Aconitic acid	μM	3.88 ± 0.09	3.61 ± 0.11	< 0.05	3.95 ± 0.15	3.51 ± 0.16	< 0.01	3.81 ± 0.11	3.71 ± 0.14	0.40	-0.47 ± 0.13	-0.12 ± 0.13	< 0.10
Cysteine glutathione disulfide	μM	1.49 ± 0.07	1.44 ± 0.10	0.65	1.54 ± 0.09	1.39 ± 0.10	< 0.10	1.43 ± 0.10	1.49 ± 0.18	0.72	-0.22 ± 0.17	-0.04 ± 0.17	0.37
Gluconic acid	μM	3.73 ± 0.10	3.70 ± 0.09	0.61	3.89 ± 0.16	3.73 ± 0.15	< 0.10	3.60 ± 0.11	3.68 ± 0.11	0.32	-0.19 ± 0.09	+0.05 ± 0.09	< 0.10
Hypotaurine	μM	1.76 ± 0.06	1.62 ± 0.06	< 0.05	1.74 ± 0.09	1.58 ± 0.08	< 0.05	1.77 ± 0.09	1.67 ± 0.10	0.28	-0.14 ± 0.10	-0.08 ± 0.10	0.69
Malic acid	μM	5.91 ± 0.26	4.88 ± 0.20	< 0.001	6.22 ± 0.52	4.40 ± 0.24	< 0.001	5.65 ± 0.19	5.29 ± 0.29	0.25	-1.89 ± 0.30	-0.36 ± 0.30	< 0.01
Methionine sulfoxide	μM	1.56 ± 0.06	1.45 ± 0.06	0.23	1.62 ± 0.08	1.42 ± 0.08	< 0.05	1.50 ± 0.09	1.48 ± 0.08	0.89	-0.24 ± 0.16	0.00 ± 0.16	0.24
Phosphorylcholine	μM	0.74 ± 0.03	0.69 ± 0.03	< 0.05	0.69 ± 0.03	0.63 ± 0.04	< 0.10	0.79 ± 0.04	0.75 ± 0.04	0.31	-0.02 ± 0.10	-0.14 ± 0.10	0.34
Pyruvic acid	μM	60.5 ± 4.20	47.53 ± 3.55	< 0.05	66.26 ± 7.10	39.92 ± 3.84	< 0.01	55.5 ± 4.80	54.13 ± 5.51	0.85	-27.07 ± 7.18	-1.64 ± 7.18	< 0.05
Succinic acid	μM	5.89 ± 0.19	5.01 ± 0.18	< 0.001	6.08 ± 0.25	4.84 ± 0.25	< 0.0001	5.72 ± 0.27	5.16 ± 0.27	0.16	-1.03 ± 0.43	-0.75 ± 0.43	0.63

Values show the means ± standard errors (SEs). The change value was calculated by subtracting the baseline value measured before the training intervention from the value measured after the training intervention. Statistical analyses were performed with two-sided paired *t* tests for the intragroup difference, [a]two-sided independent *t* tests for the intergroup difference, and [b]Wilcoxon signed-rank sum tests for the intragroup difference in the FI value.
AT, aerobic training; FI, fluorescence intensity; RT, resistance training

Extended Data 6. Blood factors that increased significantly only after aerobic training

Blood factor	Unit	All (*n* = 56)			AT (*n* = 26)			RT (*n* = 30)			Change (Δ)		
		Before	After	*p value*	Before	After	*p value*	Before	After	*p value*	AT	RT	*p value* [a]
25(OH)VitaminD	ng/mL	37.80 ± 11.89	32.09 ± 3.07	0.64	24.51 ± 2.88	34.09 ± 4.94	< 0.05	49.32 ± 22.02	30.36 ± 3.86	0.40	+9.59 ± 22.37	-18.96 ± 22.37	0.24
Myonectin	ng/mL	2.03 ± 0.13	2.14 ± 0.13	< 0.01	1.86 ± 0.06	2.01 ± 0.05	< 0.01	2.17 ± 0.23	2.26 ± 0.24	0.16	+0.14 ± 0.06	+0.09 ± 0.06	0.47
IL-15	FI[b]	-7.93 ± 1.06	-6.32 ± 1.25	< 0.05	-8.41 ± 1.78	-6.48 ± 1.67	< 0.05	-7.52 ± 1.25	-6.18 ± 1.86	0.43	+1.93 ± 0.94	+1.34 ± 1.19	0.70
MPIF-1/CCL23	pg/mL	146.02 ± 11.61	158.21 ± 13.35	< 0.05	146.44 ± 17.69	160.84 ± 19.73	< 0.05	145.65 ± 15.60	155.94 ± 18.44	0.16	+14.41 ± 7.12	+10.28 ± 7.12	0.67
CD163	ng/mL	94.57 ± 5.13	98.88 ± 5.37	0.12	92.11 ± 7.80	103.46 ± 7.35	< 0.001	96.70 ± 6.89	94.91 ± 7.78	0.67	+11.34 ± 4.13	-1.79 ± 4.13	< 0.05
Chitinase 3-like 1	ng/mL	15.42 ± 1.05	15.68 ± 1.10	0.67	14.71 ± 1.07	17.75 ± 1.94	< 0.10	16.03 ± 1.73	13.83 ± 1.07	0.22	+3.04 ± 1.63	-2.66 ± 1.63	< 0.05
IL-6Ra	ng/mL	9.03 ± 0.40	9.47 ± 0.43	0.12	8.36 ± 0.61	9.48 ± 0.49	< 0.01	9.62 ± 0.53	9.47 ± 0.69	0.71	+1.12 ± 0.39	-0.14 ± 0.39	< 0.05
IL-12(p70)	FI[b]	-2.85 ± 0.42	-2.70 ± 0.38	0.33	-2.90 ± 0.85	-2.50 ± 0.66	< 0.10	-2.80 ± 0.29	-2.87 ± 0.44	0.63	+0.40 ± 0.43	-0.07 ± 0.43	0.45
IL-34	FI[b]	-25.17 ± 1.49	-23.83 ± 1.53	< 0.01	-27.03 ± 2.87	-23.9 ± 2.62	< 0.05	-23.56 ± 1.24	-23.76 ± 1.79	0.24	+3.13 ± 1.59	-0.20 ± 1.59	< 0.10
IL-35	FI[b]	-7.21 ± 1.84	-5.75 ± 2.14	< 0.10	-8.15 ± 3.53	-3.84 ± 2.93	< 0.05	-6.39 ± 1.63	-7.41 ± 3.10	0.66	+4.31 ± 2.49	-1.02 ± 2.49	0.11
MMP-2	ng/mL	80.10 ± 3.85	92.00 ± 5.09	< 0.01	79.61 ± 5.51	93.72 ± 5.84	< 0.0001	80.52 ± 5.46	90.50 ± 8.14	0.13	+14.11 ± 6.33	+9.98 ± 6.33	0.58
Pentraxin-3	ng/mL	11.71 ± 0.75	12.87 ± 0.79	< 0.10	11.87 ± 1.37	13.02 ± 1.18	< 0.10	11.57 ± 0.76	12.75 ± 1.08	0.22	+1.15 ± 0.94	+1.18 ± 0.94	0.98
TSLP	FI[b]	10.45 ± 1.10	11.54 ± 1.23	< 0.10	12.02 ± 2.19	13.87 ± 2.16	< 0.10	9.08 ± 0.76	9.52 ± 1.24	0.46	+1.85 ± 1.15	+0.43 ± 1.15	0.34
TWEAK/TNFSF12	pg/mL	354.66 ± 15.48	371.33 ± 13.17	0.13	347.80 ± 28.34	392.14 ± 17.64	< 0.05	360.61 ± 15.68	352.68 ± 18.94	0.63	+44.34 ± 18.13	-19.69 ± 18.13	< 0.05
IL-33	FI[b]	-4.89 ± 0.27	-4.76 ± 0.27	0.42	-4.81 ± 0.36	-4.37 ± 0.35	< 0.10	-4.97 ± 0.40	-5.10 ± 0.39	0.38	+0.44 ± 0.16	-0.13 ± 0.16	< 0.05
Erythropoetin	FI[b]	-7.46 ± 0.80	-7.43 ± 0.81	0.63	-8.27 ± 1.14	-7.62 ± 1.19	< 0.05	-6.75 ± 1.12	-7.27 ± 1.11	0.23	+0.65 ± 0.45	-0.52 ± 0.45	< 0.05
FSH	mIU/mL	33.66 ± 5.24	43.88 ± 7.27	< 0.01	38.86 ± 8.03	48.57 ± 10.25	< 0.05	28.85 ± 6.82	39.37 ± 10.41	0.11	+11.2 ± 5.68	+9.47 ± 5.68	0.81
MIA	pg/mL	846.98 ± 44.15	891.83 ± 44.99	< 0.01	816.52 ± 61.96	887.18 ± 66.38	< 0.01	873.38 ± 63.09	895.86 ± 62.23	0.26	+70.66 ± 19.42	+22.48 ± 19.42	0.12
Park7/DJ-1	ng/mL	11.56 ± 0.57	12.25 ± 0.73	0.33	10.60 ± 0.49	12.59 ± 1.34	< 0.10	12.38 ± 0.96	11.95 ± 0.73	0.61	+1.99 ± 0.84	-0.43 ± 0.84	< 0.10
1-Methylnicotinamide	μM	0.32 ± 0.02	0.40 ± 0.02	< 0.01	0.30 ± 0.03	0.45 ± 0.04	< 0.01	0.33 ± 0.03	0.35 ± 0.03	0.56	+0.22 ± 0.09	+0.09 ± 0.09	0.24
ADMA	μM	0.35 ± 0.01	0.37 ± 0.01	0.11	0.34 ± 0.01	0.38 ± 0.01	< 0.05	0.36 ± 0.01	0.36 ± 0.01	0.83	+0.30 ± 0.09	+0.01 ± 0.09	< 0.05
Guanidinosuccinic acid	μM	0.34 ± 0.02	0.37 ± 0.02	< 0.05	0.30 ± 0.03	0.35 ± 0.04	< 0.10	0.37 ± 0.03	0.38 ± 0.03	0.35	+0.12 ± 0.08	+0.12 ± 0.08	1.00
His	μM	131.5 ± 2.26	133.6 ± 2.20	0.23	129.49 ± 3.27	133.50 ± 3.12	< 0.10	133.25 ± 3.15	133.69 ± 3.14	0.87	+2.98 ± 2.56	+0.79 ± 2.56	0.52
Homoarginine	μM	1.84 ± 0.07	2.04 ± 0.10	< 0.05	1.91 ± 0.13	2.15 ± 0.17	< 0.05	1.78 ± 0.08	1.94 ± 0.12	0.16	+0.29 ± 0.12	+0.18 ± 0.12	0.51
Lys	μM	342.53 ± 7.10	356.24 ± 8.33	0.14	341.98 ± 10.46	370.27 ± 14.35	< 0.10	343.01 ± 9.83	344.07 ± 8.99	0.92	+23.07 ± 9.99	-4.00 ± 9.99	0.12
Mucic acid	μM	4.47 ± 0.23	4.90 ± 0.22	< 0.05	4.42 ± 0.32	4.97 ± 0.36	< 0.10	4.51 ± 0.34	4.84 ± 0.27	0.19	+0.60 ± 0.25	+0.32 ± 0.25	0.48
N1-Methyl-4-pyridone-5-carboxamide	μM	0.31 ± 0.01	0.36 ± 0.02	< 0.05	0.28 ± 0.02	0.36 ± 0.02	< 0.05	0.33 ± 0.02	0.35 ± 0.03	0.41	+0.17 ± 0.08	+0.03 ± 0.08	0.22
Ornithine	μM	65.49 ± 1.64	68.52 ± 2.30	0.18	67.96 ± 2.03	74.73 ± 4.18	< 0.10	63.34 ± 2.48	63.13 ± 1.89	0.94	+5.04 ± 2.57	+0.14 ± 2.57	0.25

Values show the means ± standard errors (SEs). The change value was calculated as delta (Δ) by subtracting the baseline value measured before training intervention from the value measured after training intervention. Statistical analyses were performed with two-sided paired *t* tests for the intragroup difference, [a]two-sided independent *t* tests for the intergroup difference, and [b]Wilcoxon signed-rank sum tests for the intragroup difference in the FI value.
AT, aerobic training; FI, fluorescence intensity; IU, international unit; RT, resistance training

Extended Data 7. Blood factors that decreased significantly only after resistance training

Blood factor	Unit	All (n = 56)			AT (n = 26)			RT (n = 30)			Change (Δ)		
		Before	After	*p value*	Before	After	*p value*	Before	After	*p value*	AT	RT	*p value*[a]
MCP-3	FI[b]	-3.34 ± 0.55	-3.62 ± 0.56	0.11	-2.48 ± 0.76	-2.63 ± 0.74	0.64	-4.09 ± 0.79	-4.47 ± 0.82	< 0.10	-0.15 ± 0.32	-0.39 ± 0.32	0.60
MIG	pg/mL	185.63 ± 61.71	117.34 ± 9.35	0.25	254.77 ± 132.52	130.6 ± 18.32	0.34	125.71 ± 9.80	105.85 ± 6.98	< 0.05	-124.18 ± 7.54	-19.86 ± 7.54	0.38
ENA-78/CXCL5	ng/mL	1.08 ± 0.39	1.20 ± 0.52	0.51	1.46 ± 0.85	1.76 ± 1.16	0.39	0.77 ± 0.09	0.73 ± 0.09	< 0.10	+0.21 ± 0.03	-0.07 ± 0.03	0.23
Eotaxin-2/CCL24	pg/mL	63.51 ± 3.36	61.49 ± 3.36	0.34	63.92 ± 5.83	65.09 ± 5.91	0.73	63.16 ± 3.82	58.37 ± 3.64	< 0.10	+1.16 ± 2.63	-4.79 ± 2.63	0.16
I-TAC/CXCL11	pg/mL	15.79 ± 2.37	11.44 ± 1.00	< 0.10	17.83 ± 4.73	11.52 ± 1.54	0.21	14.01 ± 1.71	11.37 ± 1.33	< 0.05	-6.31 ± 1.07	-2.64 ± 1.07	0.44
MCP-2/CCL8	pg/mL	66.76 ± 11.99	48.70 ± 2.99	0.11	81.22 ± 24.51	52.41 ± 3.81	0.23	54.24 ± 6.95	45.49 ± 4.47	< 0.10	-28.81 ± 4.87	-8.75 ± 4.87	0.37
MIP-1d/CCL15	ng/mL	4.45 ± 0.36	4.00 ± 0.28	< 0.10	4.50 ± 0.49	3.94 ± 0.33	0.45	4.39 ± 0.54	4.05 ± 0.45	< 0.05	-0.69 ± 0.43	-0.17 ± 0.43	0.45
MIP-3b/CCL19	pg/mL	44.80 ± 2.65	40.50 ± 1.54	< 0.10	48.42 ± 5.08	41.69 ± 2.27	0.14	41.65 ± 2.20	39.47 ± 2.12	< 0.10	-6.73 ± 1.27	-2.19 ± 1.27	0.30
IL-17F	FI[b]	3.63 ± 0.71	3.49 ± 0.72	0.13	2.52 ± 1.06	2.48 ± 1.11	0.61	4.59 ± 0.93	4.36 ± 0.91	< 0.10	-0.04 ± 0.13	-0.23 ± 0.13	0.47
IL-23	FI[b]	-1.25 ± 0.14	-1.46 ± 0.14	0.15	-1.33 ± 0.21	-1.17 ± 0.19	0.45	-1.18 ± 0.20	-1.70 ± 0.20	< 0.01	+0.15 ± 0.19	-0.52 ± 0.19	< 0.05
IL-31	FI[b]	4.63 ± 0.76	4.46 ± 0.77	0.15	3.10 ± 1.09	3.19 ± 1.10	0.82	5.97 ± 1.00	5.57 ± 1.05	< 0.05	+0.10 ± 0.20	-0.40 ± 0.20	< 0.10
CD40LG	pg/mL	245.03 ± 11.38	191.94 ± 13.42	< 0.001	224.73 ± 15.42	200.08 ± 22.75	0.19	261.95 ± 16.02	184.87 ± 15.71	< 0.001	-16.00 ± 21.05	-77.07 ± 21.05	< 0.05
FGF21	FI[b]	-269.30 ± 12.32	-280.74 ± 10.92	< 0.001	-253.92 ± 25.00	-260.98 ± 22.46	0.20	-282.63 ± 7.59	-297.87 ± 4.82	< 0.001	-7.06 ± 5.52	-15.23 ± 5.52	0.34
CCL28	pg/mL	145.99 ± 9.40	128.75 ± 9.73	< 0.10	132.45 ± 6.87	132.88 ± 16.68	0.98	157.71 ± 16.36	125.17 ± 11.22	< 0.05	+0.43 ± 12.61	-32.54 ± 12.61	< 0.10
ANGPTL3	ng/mL	45.71 ± 2.61	43.46 ± 2.71	0.14	45.47 ± 4.15	44.08 ± 4.50	0.59	45.91 ± 3.35	42.92 ± 3.31	< 0.10	-1.38 ± 1.74	-3.00 ± 1.74	0.60
GDNF	pg/mL	4.36 ± 0.20	4.11 ± 0.19	< 0.05	4.38 ± 0.34	4.19 ± 0.29	0.23	4.34 ± 0.23	4.04 ± 0.24	< 0.10	-0.19 ± 0.16	-0.30 ± 0.16	0.62
D-Dimer + FDPs	ng/mL	2.83 ± 0.33	2.01 ± 0.17	< 0.05	1.85 ± 0.13	2.01 ± 0.28	0.54	3.65 ± 0.55	2.01 ± 0.22	< 0.01	+0.23 ± 0.56	-1.63 ± 0.56	< 0.01
Reg3A	ng/mL	6.78 ± 0.49	6.39 ± 0.43	< 0.10	6.70 ± 0.68	6.48 ± 0.58	0.49	6.85 ± 0.71	6.32 ± 0.64	< 0.05	-0.23 ± 0.24	-0.53 ± 0.24	0.44
Progranulin/PGRN	ng/mL	37.23 ± 2.11	33.68 ± 1.05	< 0.05	38.5 ± 4.09	33.06 ± 1.91	0.13	36.14 ± 1.79	34.21 ± 1.07	< 0.05	-5.44 ± 0.91	-1.93 ± 0.91	0.30
Complement Factor D/Adipsin	μg/mL	3.07 ± 0.09	2.83 ± 0.07	< 0.001	2.95 ± 0.10	2.81 ± 0.11	0.12	3.18 ± 0.13	2.85 ± 0.10	< 0.001	-0.14 ± 0.08	-0.32 ± 0.08	0.13
MSP/MST1	μg/mL	2.69 ± 0.07	2.58 ± 0.06	< 0.05	2.55 ± 0.06	2.50 ± 0.08	0.36	2.81 ± 0.12	2.65 ± 0.09	< 0.05	-0.04 ± 0.06	-0.16 ± 0.06	0.14
LBP	μg/mL	5.91 ± 0.28	5.31 ± 0.15	< 0.05	6.16 ± 0.54	5.48 ± 0.25	0.19	5.69 ± 0.24	5.16 ± 0.18	< 0.05	-0.69 ± 0.21	-0.53 ± 0.21	0.76
CXCL4/PF4	μg/mL	11.17 ± 1.22	8.82 ± 0.77	< 0.10	11.18 ± 2.18	8.30 ± 0.97	0.22	11.16 ± 1.32	9.26 ± 1.18	< 0.10	-2.87 ± 1.11	-1.90 ± 1.11	0.69
ADP	μM	1.23 ± 0.11	1.06 ± 0.09	< 0.10	0.98 ± 0.12	1.01 ± 0.14	0.64	1.45 ± 0.17	1.10 ± 0.12	< 0.10	-0.21 ± 0.20	-0.43 ± 0.20	0.38
Ethanolamine phosphate	μM	4.15 ± 0.20	3.46 ± 0.18	< 0.01	3.77 ± 0.29	3.29 ± 0.29	0.14	4.47 ± 0.26	3.61 ± 0.23	< 0.05	-0.48 ± 0.33	-0.91 ± 0.33	0.34
N,N-Dimethylglycine	μM	6.95 ± 0.42	6.53 ± 0.46	< 0.10	7.71 ± 0.80	7.34 ± 0.92	0.40	6.29 ± 0.32	5.83 ± 0.27	< 0.05	-0.39 ± 0.21	-0.45 ± 0.21	0.90
Nicotinamide	μM	0.35 ± 0.02	0.30 ± 0.01	< 0.05	0.32 ± 0.02	0.29 ± 0.02	0.15	0.37 ± 0.02	0.32 ± 0.02	< 0.05	-0.10 ± 0.08	-0.10 ± 0.08	0.98

Values show the means ± standard errors (SEs). The change value was calculated as delta (Δ) by subtracting the baseline value measured before the training intervention from the value measured after the training intervention. Statistical analyses were performed with two-sided paired t tests for the intragroup difference, [a]two-sided independent t tests for the intergroup difference, and [b]Wilcoxon signed-rank sum tests for the intragroup difference in the FI value.
AT, aerobic training; FI, fluorescence intensity; RT, resistance training

Extended Data 8. Blood factors that increased significantly only after resistance training

Blood factor	Unit	All (*n* = 56)			AT (*n* = 26)			RT (*n* = 30)			Change (Δ)		
		Before	After	*p value*	Before	After	*p value*	Before	After	*p value*	AT	RT	*p value* [a]
Cathepsin B	ng/mL	136.58 ± 5.32	144.99 ± 5.85	< 0.10	134.41 ± 8.34	139.60 ± 8.01	0.43	138.46 ± 6.93	149.67 ± 8.46	< 0.10	+5.20 ± 6.27	+11.21 ± 6.27	0.51
IL-8/CXCL8	pg/mL	8.30 ± 0.79	9.25 ± 0.81	< 0.10	9.50 ± 1.50	9.74 ± 1.40	0.64	7.25 ± 0.69	8.83 ± 0.94	< 0.05	-0.14 ± 0.76	+1.59 ± 0.76	0.17
RANTES	ng/mL	12.23 ± 0.37	12.53 ± 0.44	< 0.01	12.25 ± 0.58	12.32 ± 0.58	0.16	12.20 ± 0.49	12.72 ± 0.65	< 0.05	+0.06 ± 0.47	+0.52 ± 0.47	0.46
TNF-β	pg/mL	143.00 ± 6.35	145.29 ± 4.71	0.48	151.35 ± 13.36	148.18 ± 9.49	0.54	135.77 ± 2.36	142.78 ± 3.28	< 0.10	-3.16 ± 3.92	+7.01 ± 3.92	0.12
α1-Microglobulin	ng/mL	11.73 ± 0.18	12.00 ± 0.20	< 0.05	11.77 ± 0.33	11.90 ± 0.35	0.46	11.7 ± 0.19	12.08 ± 0.21	< 0.05	+0.13 ± 0.14	+0.38 ± 0.14	0.27
BMP-7	pg/mL	78.98 ± 5.14	87.67 ± 7.18	< 0.05	84.41 ± 9.46	95.33 ± 13.55	0.10	74.28 ± 5.01	81.03 ± 6.48	< 0.10	+10.92 ± 3.44	+6.75 ± 3.44	0.56
FABP4/A-FABP	ng/mL	7.73 ± 1.51	8.04 ± 1.29	0.62	9.51 ± 3.01	9.06 ± 2.31	0.72	6.19 ± 1.07	7.16 ± 1.34	< 0.05	-0.45 ± 0.44	+0.98 ± 0.44	0.26
CTRP9/C1qTNF9	pg/mL	222.84 ± 68.97	211.65 ± 48.98	0.60	303.27 ± 146.19	263.90 ± 102.28	0.39	153.13 ± 22.69	166.36 ± 22.94	< 0.01	-39.37 ± 4.71	+13.22 ± 4.71	0.22
FGF-23	pg/mL	31.60 ± 8.92	33.89 ± 9.46	0.28	31.44 ± 13.06	29.22 ± 11.42	0.42	31.74 ± 12.42	37.93 ± 14.78	< 0.05	-2.23 ± 2.95	+6.20 ± 2.95	< 0.05
FGF-13	FI[b]	51.37 ± 1.01	52.74 ± 1.11	< 0.05	52.04 ± 1.94	52.50 ± 2.20	0.79	50.78 ± 0.89	52.95 ± 0.87	< 0.001	+0.46 ± 0.60	+2.17 ± 0.60	0.28
VEGFR1/Flt-1	pg/mL	130.29 ± 5.48	136.83 ± 5.53	< 0.05	125.51 ± 9.50	130.07 ± 9.12	0.35	134.43 ± 6.13	142.70 ± 6.60	< 0.10	+4.55 ± 4.38	+8.27 ± 4.38	0.57
PlGF	FI[b]	50.21 ± 1.61	49.89 ± 1.62	0.29	52.42 ± 2.47	49.81 ± 2.64	0.39	48.28 ± 2.08	49.97 ± 2.02	< 0.05	-2.62 ± 0.81	+1.68 ± 0.81	< 0.05
2-Aminoadipic acid	μM	0.69 ± 0.03	0.74 ± 0.03	0.12	0.75 ± 0.05	0.79 ± 0.04	0.64	0.63 ± 0.03	0.70 ± 0.04	< 0.01	+0.00 ± 0.09	+0.07 ± 0.09	0.64
3-(4-Hydroxyphenyl)propionic acid	μM	0.86 ± 0.11	0.90 ± 0.11	0.98	0.97 ± 0.20	0.76 ± 0.15	0.30	0.75 ± 0.09	1.08 ± 0.15	< 0.05	-0.16 ± 0.15	+0.04 ± 0.15	0.47
Creatinine	μM	89.81 ± 1.58	93.88 ± 1.86	< 0.01	89.71 ± 2.23	91.89 ± 2.81	0.27	89.89 ± 2.27	95.61 ± 2.48	< 0.01	+2.51 ± 1.85	+5.97 ± 1.85	0.19
Erythrose 4-phosphate	μM	8.51 ± 0.85	9.25 ± 0.53	0.61	9.80 ± 1.75	9.03 ± 0.74	0.92	7.41 ± 0.43	9.50 ± 0.76	< 0.10	+1.61 ± 1.30	+1.47 ± 1.30	0.95
Ethanolamine	μM	5.67 ± 0.10	5.97 ± 0.12	< 0.05	5.66 ± 0.15	5.89 ± 0.16	0.18	5.68 ± 0.14	6.05 ± 0.16	< 0.10	+0.22 ± 0.20	+0.39 ± 0.20	0.53
Homovanillic acid	μM	1.42 ± 0.06	1.57 ± 0.07	< 0.05	1.51 ± 0.09	1.46 ± 0.11	0.65	1.35 ± 0.06	1.65 ± 0.09	< 0.0001	+0.07 ± 0.08	+0.40 ± 0.08	< 0.05
Ile	μM	91.03 ± 1.78	93.20 ± 1.58	0.23	93.53 ± 2.57	92.59 ± 2.50	0.71	88.87 ± 2.43	93.73 ± 2.04	< 0.10	-1.26 ± 2.49	+4.55 ± 2.49	0.10
Imidazolelactic acid	μM	0.22 ± 0.01	0.24 ± 0.01	< 0.05	0.22 ± 0.01	0.23 ± 0.01	0.30	0.23 ± 0.01	0.25 ± 0.01	< 0.05	+0.06 ± 0.08	+0.13 ± 0.08	0.57
Isethionic acid	μM	2.01 ± 0.06	2.12 ± 0.05	< 0.05	2.05 ± 0.09	2.13 ± 0.06	0.25	1.97 ± 0.08	2.11 ± 0.08	< 0.05	+0.08 ± 0.10	+0.18 ± 0.10	0.43
Isobutyrylcarnitine	μM	0.13 ± 0.01	0.15 ± 0.01	< 0.05	0.14 ± 0.01	0.14 ± 0.01	0.50	0.13 ± 0.01	0.15 ± 0.01	< 0.05	+0.07 ± 0.09	+0.16 ± 0.09	0.48
Isovalerylcarnitine	μM	0.05 ± 0.00	0.05 ± 0.00	< 0.05	0.05 ± 0.01	0.05 ± 0.00	0.11	0.05 ± 0.00	0.05 ± 0.00	< 0.10	+0.21 ± 0.10	+0.18 ± 0.10	0.83
Leu	μM	176.04 ± 2.70	184.35 ± 2.6	< 0.01	178.98 ± 4.12	186.14 ± 4.28	0.14	173.5 ± 3.56	182.81 ± 3.18	< 0.05	+9.25 ± 3.54	+9.35 ± 3.54	0.99
Met	μM	34.2 ± 0.96	37.22 ± 0.96	< 0.05	34.89 ± 1.29	36.75 ± 1.49	0.21	33.60 ± 1.41	37.62 ± 1.25	< 0.05	+2.55 ± 1.67	+2.52 ± 1.67	0.99
N1-Acetylspermidine	μM	0.06 ± 0.00	0.07 ± 0.00	0.18	0.06 ± 0.00	0.06 ± 0.00	0.75	0.06 ± 0.00	0.07 ± 0.00	< 0.10	+0.14 ± 0.08	+0.07 ± 0.08	0.54
N6-Methyllysine	μM	3.71 ± 0.31	3.87 ± 0.36	0.15	3.87 ± 0.52	3.90 ± 0.60	0.89	3.57 ± 0.36	3.84 ± 0.44	< 0.05	+0.08 ± 0.12	+0.33 ± 0.12	0.29
Pantothenic acid	μM	0.32 ± 0.02	0.33 ± 0.02	< 0.10	0.33 ± 0.03	0.33 ± 0.04	0.31	0.30 ± 0.03	0.33 ± 0.03	< 0.10	-0.02 ± 0.09	+0.16 ± 0.09	0.14
Pelargonic acid	μM	1.17 ± 0.03	1.23 ± 0.02	< 0.05	1.21 ± 0.04	1.27 ± 0.03	0.24	1.13 ± 0.04	1.21 ± 0.03	< 0.10	+0.06 ± 0.08	+0.11 ± 0.08	0.64
Phe	μM	118.42 ± 3.22	118.15 ± 1.56	0.92	124.15 ± 6.26	115.98 ± 2.74	0.12	113.46 ± 2.36	120.02 ± 1.67	< 0.05	-4.10 ± 2.42	+5.99 ± 2.42	< 0.01
Quinic acid	μM	1.95 ± 0.21	2.20 ± 0.26	0.26	2.35 ± 0.40	1.99 ± 0.35	0.33	1.60 ± 0.16	2.39 ± 0.37	< 0.05	-0.19 ± 0.36	+0.74 ± 0.36	< 0.10
Trigonelline	μM	1.20 ± 0.19	1.54 ± 0.20	< 0.10	1.56 ± 0.35	1.76 ± 0.39	0.54	0.90 ± 0.15	1.35 ± 0.17	< 0.05	+0.25 ± 0.21	+0.47 ± 0.21	0.58
Uridine	μM	16.28 ± 0.34	17.07 ± 0.35	< 0.01	15.67 ± 0.44	16.39 ± 0.30	0.11	16.82 ± 0.49	17.67 ± 0.58	< 0.05	+0.78 ± 0.43	+0.83 ± 0.43	0.93
Urocanic acid	μM	0.25 ± 0.09	0.21 ± 0.02	0.76	0.37 ± 0.19	0.22 ± 0.03	0.48	0.15 ± 0.02	0.21 ± 0.03	< 0.10	+0.06 ± 0.06	+0.23 ± 0.06	0.16

Values show the means ± standard errors (SEs). The change value was calculated by subtracting the baseline value measured before the training intervention from the value measured after the training intervention. Statistical analyses were performed with two-sided paired *t* tests for the intragroup difference, [a]two-sided independent *t* tests for the intergroup difference, and [b]Wilcoxon signed-rank sum tests for the intragroup difference in the FI value.

AT, aerobic training; FI, fluorescence intensity; RT, resistance training

Supplementary Table 1. Circulating factors measured in this study

Measurement Panel/Method	Catalog No.	Measured circulating factors (>1,487)										Protocol Ref.
Bio-Plex Cytokine panel	# F-12007283	β-NGF	CTACK	Eotaxin	FGF basic	G-CSF	GM-CSF	GRO-α	HGF	IL-1α	IL-1β	http://www.bio-rad.com/webroot/web/pdf/lsr/literature/10031990.pdf
		IL-1Ra	IL-2Ra	IL-3	IL-4	IL-5	IL-6	IL-7	IL-9	IL-13	IL-15	
		IL-16	IL-17A	IL-18	IP-10	LIF	M-CSF	MCP-1(MCAF)	MCP-3	MIF	MIG	
		MIP-1α	MIP-1β	PDGF-BB	RANTES	SCF	SCGF-β	SDF-1α	TNF-α	TNF-β	TRAIL	
		VEGF										
Bio-Plex Chemokine panel	# F-171-AK99MR2-20	6Ckine/CCL21	BCA-1/CXCL13	ENA-78/CXCL5	Eotaxin-2/CCL24	Eotaxin-3/CCL26	Fractalkine/CX3CL1	GCP-2/CXCL6	Gro-β/CXCL2	I-309/CCL1	I-TAC/CXCL11	http://www.bio-rad.com/webroot/web/pdf/lsr/literature/10031990.pdf
		MCP-2/CCL8	MCP-4/CCL13	MDC/CCL22	MIP-1δ/CCL15	MIP-3α/CCL20	MIP-3β/CCL19	MPIF-1/CCL23	SCYB16/CXCL16	TARC/CCL17	TECK/CCL25	
Bio-Plex Inflammation panel	# F-171-AL001M	APRIL/TNFSF13	BAFF/TNFSF13B	CD163	CD30/TNFRSF8	Chitinase 3-like 1	gp130	IFN-α2	IFN-β	IFN-γ	IL-2	http://www.bio-rad.com/webroot/web/pdf/lsr/literature/10044282.pdf
		IL-6Ra	IL-8/CXCL8	IL-10	IL-11	IL-12(p40)	IL-12(p70)	IL-19	IL-20	IL-22	IL-26	
		IL-27(p28)	IL-28A/IFN-λ2	IL-29/IFN-λ1	IL-32	IL-34	IL-35	LIGHT/TNFSF14	MMP-1	MMP-2	MMP-3	
		Osteocalcin	Osteopontin	Pentraxin-3	TNF-R1	TNF-R2	TSLP	TWEAK/TNFSF12				
Bio-Plex Th17 Cytokine panel	# F-BP-HTH17-xplex07	IL-17F	IL-21	IL-23	IL-25	IL-31	IL-33	sCD40L				https://www.bio-rad.com/webroot/web/pdf/lsr/literature/10023381.pdf
Milliplex Myokine panel	# F-MIL-HMYOMAG-56K-08	Apelin	BDNF	Erythropoetin	Myostatin/GDF8	FABP3	Irisin	FGF21	Osteocrin/Musclin			https://www.sigmaaldrich.com/deepweb/assets/sigmaaldrich/product/documents/394/657/protocol-hmyomag56k-mk.pdf
Milliplex Metabolic Hormone panel	# F-MIL-HMHEMAG-34K-07	Ghrelin	GIP	GLP-1	Glucagon	PP	PYY	Amylin				https://www.sigmaaldrich.com/deepweb/assets/sigmaaldrich/product/documents/259/597/protocol-hmhemag34k-mk.pdf
Milliplex Pituitary panel	# F-MIL-HPTP1MAG-66K-05	ACTH	CNTF	FSH	LH	TSH						https://www.sigmaaldrich.com/deepweb/assets/sigmaaldrich/product/documents/168/307/protocol-hptp1mag66k-mk.pdf
Milliplex Multi-Species Hormone panel	# MSHMAG-21K	Cortisol	Estradiol	Progesterone	Testosterone	T3	T4					https://www.sigmaaldrich.com/deepweb/assets/sigmaaldrich/product/documents/172/495/protocol-mshmag21k-mk.pdf
Milliplex IGF panel	# F-MIL-HIGFMAG-52K-02	IGF-I	IGF-II									https://www.sigmaaldrich.com/deepweb/assets/sigmaaldrich/product/documents/286/632/protocol-higfmag-52k-mk.pdf
Lumines LXSAH	# LXSAHM-39	ANGPTL6	Insulin	α1-Microglobulin	Galectin-9	C-Peptide	S100A8	IL-4Rα	EGF	Angiopoietin-2	FLRG	https://resources.rndsystems.com/pdfs/datasheets/lxsah.pdf
		BMP-7	Calbindin D	NT-Pro-ANP	Prolactin	IGFBP-1	Procalcitonin	AgRP/ART	CCL28	PCSK9	S100A9	
		TRANCE/TNFSF11/RANKL	PDGF-CC	IGFBP-4	PDGF-DD	B7-H3	Relaxin-2	TIM-1\KIM-1\HAVCR	Synuclein-α	MIA	Midkine	
		GDF-15	α-Fetoprotein	Chemerin	ANGPTL3	ANGPTL4	CD40 Ligand\TNFSF5	TRACP/PAP/ACP5	Flt-3 Ligand	GDNF		
	# LXSAHM-31	VEGFR2/KDR/Flk-1	NT-4	PBEF/Visfatin	MMP-7	S100B	FABP4/A-FABP	MMP-8	Syndecan-1/CD138	Dkk-1	CTRP9/C1qTNF9	
		MMP-12	Cathepsin S	Park7/DJ-1	D-Dimer+FDPs	Growth hormone	SOST/Sclerostin	Resistin	Reg3A	NRG1-β1	FGF-23	
		Progranulin/PGRN	Renin	IL-1 R2	IFN-α	IL-36β/IL-1F8	CXCL14/BRAK	FGF acidic	CD44	Stanniocalcin 1/STC-1	Endostatin	
		IGFBP-rp1/IGFBP-7										
	# LXSAHM-18	FGF-13	Osteoprotegerin	MMP-13	VEGFR1/Flt-1	XCL1/Lymphotactin	Osteoactivin/GPNMB	Total Inhibin	IGFBP-3	Follistatin-like 1/FSTL1	VEGFR3/Flt-4	
		SP-D	IL-1 R1	NT-3	IL-28B/IFN-λ3	PlGF	VEGF-D	Serpin A12	UCH-L1/PGP95			
	# LXSAHM-11	Chitinase 3-like 1	HB-EGF	IL-27	Oncostatin M	PDGF-AB	VEGF-C	Leptin/OB	IL-17C	TGF-α	TNF R2/TNFRSF1B	
		MMP-10										
	# LXSAHM-02	Periostin/OSF-2	TNF R1/TNFRSF1A									
	# LXSAHM-17	MMP-9	BDNF	Ferritin	IGFBP-2	CCL14/HCC-1/HCC-3	CCL18/PARC	SPARC	Lactoferrin	N-Cadherin	Myoglobin	
		HMW Adiponectin	SHBG	EN-RAGE/S100A12	Lipocalin-2/NGAL	PDGF-AA	Angiopoietin-1	Cystatin C				
	# LXSAHM-03	DPP4	Complement factor D/Adipsin	TIMP-1								
	# LXSAHM-13	MSP/MST1	IGFBP-6	CD14	Component C2	Serpin E1/PAI-1	Adiponectin/Acrp30	CXCL7/NAP-2	LBP	Serpin A7/TBG	C-Reactive protein	
		Angiogenin	Properdin	Serpin A10/ZPI								
	# LXSAHM-09	ITIH4	β2-Microglobulin	Serpin C1	Protein S/PROS1	Serpin F1/PEDF	CXCL4/PF4	Fetuin A/AHSG	Retinol-binding protein 4	Serpin A4/Kallistatin		
ELISA	# ADI-900-215-0001	25(OH) Vitamin D										https://www.enzolifesciences.com/fileadmin/files/manual/ADI-900-215_insert.pdf
	# OKBB00301	Cathepsin B										https://www.avivasysbio.com/pub/media/pdf/products/OKBB00301.pdf
	# DEIA-XY2197	Myonectin										https://img2.creative-diagnostics.com/pdf/DEIA-XY2197.pdf
CE-TOFMS/LC-TOFMS		Metabolites (>1,200)										https://en.humanmetabolome.com/
Blood biochemistry		Total protein	Triglyceride	Total cholesterol	HDL cholesterol	LDL cholesterol	Albumin	Insulin				

The circulating levels of cytokines and hormones in serum were measured with the following multiplex assays: Bio-Plex (Bio-Rad Laboratories, Hercules, CA, USA), Milliplex (Merck Millipore, Burlington, MA, USA), and Luminex (R&D Systems, Minneapolis, MN, USA) and enzyme-linked immunosorbent assay (ELISA). Over 1,200 metabolites in plasma were measured with a capillary electrophoresis and liquid chromatography-mass spectrometry-based system (Dual Scan, Human Metabolome Technologies, Boston, MA, USA); the details of the instruments and measured metabolites are shown on the manufacturer website (https://en.humanmetabolome.com/).
ELISA, enzyme-linked immunosorbent assay; HDL, high-density lipoprotein; LDL, low-density lipoprotein; TOFMS, time of flight mass spectrometry

Supplementary Table 2. Dermal ECM-related genes analyzed in this study

Gene Symbol	Gene Name	TaqMan® Assay ID
COL1A1	collagen type I alpha 1	Hs00164004_m1
COL1A2	collagen type I alpha 2 chain	Hs01028956_m1
COL3A1	collagen type III alpha 1 chain	Hs00943809_m1
COL5A1	collagen type V alpha 1	Hs00609133_m1
COL6A1	collagen type VI alpha 1	Hs01095585_m1
COL12A1	collagen type XII alpha 1 chain	Hs00189184_m1
COL14A1	collagen type XIV alpha 1 chain	Hs00964045_m1
HAS2	hyaluronan synthase 2	Hs00193435_m1
HAS1	hyaluronan synthase 1	Hs00758053_m1
DCN	decorin	Hs00754870_s1
BGN	biglycan	Hs00156076_m1
VCAN	versican	Hs00171642_m1
CHPF	chondroitin polymerizing factor	Hs00226041_m1
CHSY1	chondroitin sulfate synthase 1	Hs00208704_m1
CHSY3	chondroitin sulfate synthase 3	Hs02380326_s1
ELN	elastin	Hs00355783_m1
GAPDH	glyceraldehyde-3-phosphate dehydrogenase	Hs02786624_g1

The expression level of dermal extracellular matrix-related genes listed above was analyzed by real-time PCR with the primers and probes of TaqMan® gene expression assays.
ECM, extracellular matrix; PCR, polymerase chain reaction
